# Altered expression of K13 disrupts DNA replication and repair in *Plasmodium falciparum*

**DOI:** 10.1186/s12864-018-5207-7

**Published:** 2018-11-29

**Authors:** Justin Gibbons, Katrina A. Button-Simons, Swamy R. Adapa, Suzanne Li, Maxwell Pietsch, Min Zhang, Xiangyun Liao, John H. Adams, Michael T. Ferdig, Rays H. Y. Jiang

**Affiliations:** 1 Department of Molecular Medicine, Morsani College of Medicine, University of South Florida, Tampa, USA; 2 Center for Global Health and Infectious Diseases Research, College of Public Health, University of South Florida, Tampa, USA; 3 Eck Institute for Global Health, Department of Biological Sciences, University of Notre Dame, Notre Dame, USA; 4 Department of Computer Science & Engineering, University of South Florida, Tampa, USA

**Keywords:** Malaria, Artemisinin, K13, Drug-resistance

## Abstract

**Background:**

*Plasmodium falciparum* exhibits resistance to the artemisinin component of the frontline antimalarial treatment Artemisinin-based Combination Therapy in South East Asia. Millions of lives will be at risk if artemisinin resistance (ART-R) spreads to Africa. Single non-synonymous mutations in the propeller region of PF3D7_1343700,“K13” are implicated in resistance. In this work, we use transcriptional profiling to characterize a laboratory-generated *k13* insertional mutant previously demonstrated to have increased sensitivity to artemisinins to explore the functional role of *k13*.

**Results:**

A set of RNA-seq and microarray experiments confirmed that the expression profile of *k13* is specifically altered during the early ring and early trophozoite stages of the mutant intraerythrocytic development cycle. The down-regulation of *k13* transcripts in this mutant during the early ring stage is associated with a transcriptome advance towards a more trophozoite-like state. To discover the specific downstream effect of *k13* dysregulation, we developed a new computational method to search for differential gene expression while accounting for the temporal sequence of transcription. We found that the strongest biological signature of the transcriptome shift is an up-regulation of DNA replication and repair genes during the early ring developmental stage and a down-regulation of DNA replication and repair genes during the early trophozoite stage; by contrast, the expressions of housekeeping genes are unchanged. This effect, due to *k13* dysregulation, is antagonistic, such that *k13* levels are negatively correlated with DNA replication and repair gene expression.

**Conclusion:**

Our results support a role for *k13* as a stress response regulator consistent with the hypothesis that artemisinins mode of action is oxidative stress and *k13* as a functional homolog of *Keap1* which in humans regulates DNA replication and repair genes in response to oxidative stress.

**Electronic supplementary material:**

The online version of this article (10.1186/s12864-018-5207-7) contains supplementary material, which is available to authorized users.

## Background

The World Health Organization estimates that malaria killed 429,000 people, mostly children under the age of 5 in 2015 [1]. Prior to Artemisinin-based combination therapy (ACT) becoming the World Health Organization recommend treatment for uncomplicated *Plasmodium falciparum* infection, approximately 1,000,000 people were being killed by malaria annually [2]. It has been estimated that widespread ACT resistance would lead to more than 116,000 additional malaria deaths each year [3]. ACT resistant *P. falciparum* is already present in South East Asia with the ACT dihydroartemisinin-piperaquine having treatment failure rates as high as 46% in the Pursat province of Cambodia [4]. Alarmingly, resistant strains are reported to have spread to Thailand, Laos and Vietnam [5, 6].

Multiple lines of evidence suggest that the resistance mechanism involves pausing parasite development in the ring stage, which is less susceptible to artemisinin, in response to drug treatment [7–9]. Consistent with this observation, artesunate treatment has been reported to stimulate entry into a latent developmental state due to PK4 phosphorylation of eIF2α [10]; furthermore, resistant clinical isolates show an up-regulated protein folding response and down-regulation of the DNA replication machinery with a delayed progression out of the ring stage [11]. Resistant strains created in vitro by drug selection demonstrate altered gene expression in oxidative stress, protein damage, and cell cycle pathways [12].

The gene with the strongest association with artemisinin resistance is *k13* [13–16]. Crystal structure similarity suggests that K13 is a homolog of the human E3 ubiquitin substrate adaptor Keap1 with a root-mean-square deviation between the propeller domains (4zgc and 1u6d) of 1.298 Å. Evidence from Mbengue et al. [17] suggests that K13 plays a role in regulating ubiquitination. This human homolog of K13 is a well-characterized transcriptional regulator of oxidative stress response [18], but the processes regulated by K13 remain unknown in malarial parasites. Because *k13* is likely essential [19, 20] knocking out its function is not an experimental option and regulatory mutants provide a path to decipher K13’s function. Birnbaum et al. [19] reported that conditionally knocking out *k13* halts growth after 3 days at the ring stage, but the mechanisms underlying *k13* essentiality are unknown.

In this work, we report on a *k13* dysregulated mutant (PB58) [20, 21]. Previous studies using standard 72 h growth inhibition assays showed the mutant to be more sensitive than the parent NF54 strain to artemisinins (artesunate, artelinic acid, artemether, artemisinin, dihydroartemisinin; See Additional file 1: S2) and the proteasome inhibitor Bortezomib [20, 21]. The increased sensitivity to a proteasome inhibitor is interesting because the resistance to ACT has been linked to the ubiquitin/proteasome system [17, 22]. This mutant carries a single transposon insertion in the 5’ UTR of *k13* in the NF54 background. Studies utilizing QISeq verified the absence of other changes in the genomic background [23, 24]. Given K13’s BTB and propeller domains structural similarity to the transcriptional regulator Keap1, we hypothesized that dysregulation of K13 will result in an altered transcriptome of functionally-connected genes. Therefore, we conducted RNA-seq on various stages of the intraerythrocytic developmental cycle (IDC) to understand the cellular processes regulated by *k13*.

## Results

### Validation of specific K13 dysregulation in the mutant

**Fig. 1:**
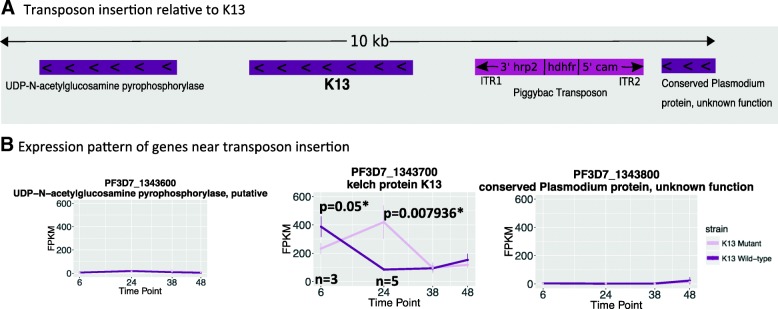
Transposon insertion and its effect on gene expression. (**a**) Insertion of a PiggyBac Transposon in the 5′ upstream region the gene *k13* (**b**) results in the gene being aberrantly down-regulated at the 6 h time point and up-regulated at the 24 h time point. The expression of *k13*’s same strand neighbors are unaffected by the insertion. The changes in K13 expression are consistent with the known regulation of the calmodulin promoter in *P. falciparum*. The transcript expression is measured in fragments per kilobase per million mapped reads (FPKM). The abbreviations for the piggybac transposon are: inverted terminal repeat 1 (ITR1), *histidine-rich protein-2 (hrp2),* human *dihydrofolate reductase* (hdhfr), regulatory elements of *calmodulin* (cam), and inverted terminal repeat 2 (ITR2). The insertion occurs 1034 nucleotides up-stream of *k13* (see Additional file 1: S1 for a finer resolution mapping of the insertion site)

The *k13* mutant carries a *piggyBac* transposon insertion in the promoter region (Fig. 1a) as previously reported by Pradhan, Siwo et al. [21]. As shown in Fig. 1a there are two other genes next to *k13* on the same DNA strand. Figure 1b shows the expression levels of the genes immediately flanking *k13* are unaffected by the transposon insertion, whereas *k13* expression is significantly altered at 6 and 24 h of the IDC (*p*-values of 0.05 and 0.007936 from Wilcoxon rank sum test with p-values corrected using the Holm method, respectively). Thus in the mutant *k13* is down-regulated during the early ring stage and is up-regulated during the early trophozoite stage, as compared to its wild-type parental strain NF54. Microarray measurements from these same time points are consistent with this interpretation (Additional file 1: Figure S3).

### *K13* mutant transcriptome is overall simliar to wild-type with the exception of several biological processes

**Fig. 2:**
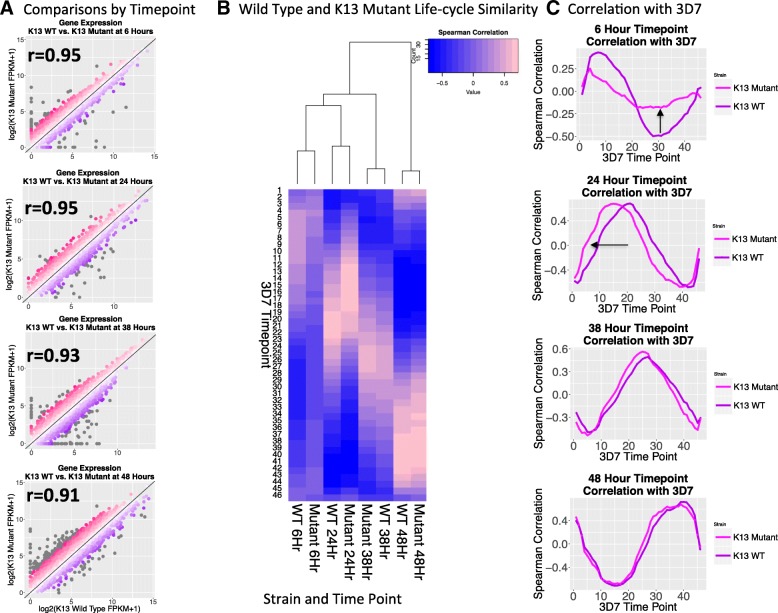
Effects of K13 dysregulation on transcriptome. (**a**) Comparing wild-type and K13 mutant transcript expression at their respective time points shows that the two strains are very similar. Grey dots represent absolute fold changes greater than 2.5. (**b**) Clustering all of the time-points based on their similarity to the 3D7 reference transcriptome from Derisi shows that from a global perspective the two strains are very similar and the time points have their highest similarities to the expected reference time-points and progression through the erythrocytic cycle is visible in the heatmap. However, at the 6 h time-point the mutant strain shows a stronger similarity to trophozoite time-points compared to the wild-type strain and at 24 h the mutant shows a less dramatic shift towards similarity with earlier time points. (**c**) Plotting the sample correlations with the 3D7 reference transcriptome from Derisi makes the disruption to the mutant 6 h transcriptome more evident. With the exception of the mutant 6 h they all show that there is a gradual increasing in similarity as the sample time point approaches the equivalent 3D7 time point and then a gradual decrease in similarity as it moves away from the time point. This periodic structure is disrupted in the mutant at 6 h. The sequencing quality of the wild-type and mutant 6 h samples are equivalent (Additional file 1: S1) indicating this disruption cannot be attributed to differences in sequencing quality. This transcriptomic shift is also not attributable to increased sample variation since variation in the 6 h samples is no greater than the variation of the other time point samples (Additional file 1: S5) or to a transposon specific effect (Additional file 1: S6). The arrows are indicating the direction of the transcriptome shifts

Our initial global transcriptome analysis showed overall conserved patterns of gene expression between the mutant and wild-type transcriptomes. Even at the time points where *k13* is dysregulated, the transcriptomes correlate well between the wild-type and mutant strains (Pearson’s r of 0.95 for both 6 and 24 h) (Fig. 2a). Next we analyzed the developmental time points of these transcriptomes by using a previously published study with extensive time points [25]. When the samples are clustered based upon their similarity to the Derisi 3D7 reference transcriptome [27], the wild-type and mutant strains of the same time point show the same relationships to the 3D7 reference time points and progression through the IDC, which is evident in the heatmap for both the wild-type and mutant transcriptomes (Fig. 2b). However, compared with the wild-type the mutant strain at 6 h does not show as strong of negative correlations with the trophozoite stage time points as the wild-type strain; and this pattern becomes even more evident when the correlations to the 3D7 reference IDC are plotted out as line graphs (Fig. 2c).

The IDC is cyclical with the majority of genes showing sinusoidal expression [25]. When the correlation of a transcription profile for a single time point against the reference transcriptome of 3D7 is plotted there will be an increase in the correlation coefficient as the sample time point approaches the corresponding reference time point, followed by a steady decline in the correlation coefficient as the sample time point becomes more distant to the reference time point until a new inflection point is reached. If the transcription profile of the mutant line is out of synch with normal IDC patterns this curve will not be smooth.

As shown in Fig. 2c, at 6 h in the *k13* mutant, the transcriptional rhythms are no longer in phase, suggesting that a disruption in transcriptional regulation has occurred that advanced the 6-h transcriptome towards a more trophozoite-like state. The sequencing quality for the wild-type and mutant 6-h samples are equivalent (Additional file 1: S4), indicating the disruption seen in the mutant at 6 h cannot be attributed to library preparation differences. Further, variation between biological replicates at 6 h is not significantly different than variation at the other time point samples (*p*-values > 0.34 by Wilcoxon rank sum test; Additional file 1: S5), indicating increased sample variability is also not responsible for the observed IDC correlation plot distortion. Disruptions to IDC correlation plots are also not present in a *piggyBac* mutant with an insertion in gene PF3D7_1305500 (Additional file 1: S6), indicating the transposon does not cause cell-cycle shifts; and the effect is specific to the *k13* mutant. The fact that the 6-h samples display the greatest divergence is particularly puzzling because differential expression analysis with EdgeR shows that the 6-h samples have the fewest number of differentially expressed genes (Additional file 1: S7). Given that the sequencing data have good quality scores (Additional file 1: S4), the variation between the 6-h replicates is not unusually high and the paucity of differentially expressed genes suggests that the observed shift in the transcriptional rhythms at 6-h may be due to a small but consistent shift in the expression levels of stage specific genes functionally linked to *k13*.

**Fig. 3:**
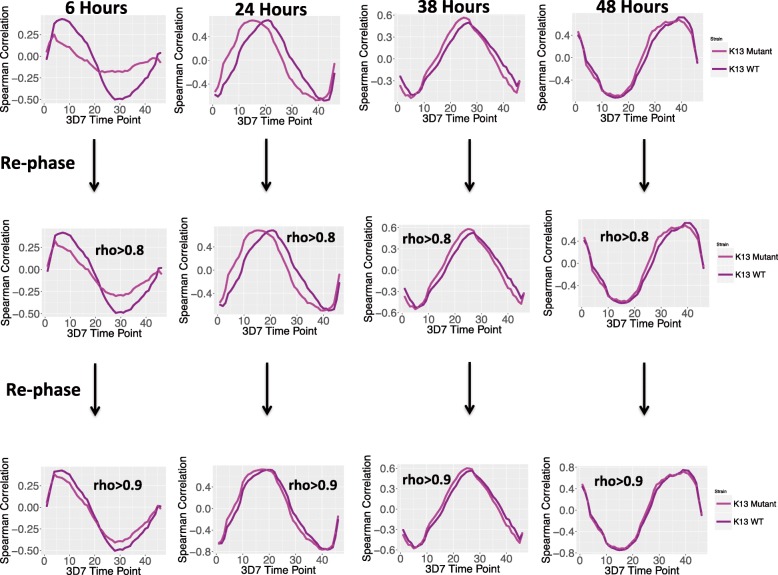
The DI Algorithm identifies genes responsible for IDC correlation shifts. The DI algorithm identifies the genes responsible for decreasing the correlations of the mutant and wild-type transcriptomes by iteratively removing genes that show the largest changes in rank expression between them. The genes are removed in one quantile batches and the correlations between the transcriptomes are re-computed. The filtering process ends when the correlation between the mutant and wild-type transcriptomes is at least as good as the highest correlation either has with the Derisi 3D7 IDC transcriptome. The correlation to Derisi 3D7 IDC transcriptome was chosen as an unbiased cut-off since 2 samples from the same lab should be at least as well correlated with each other as a sample from a different lab. For the 6 h samples the DI algorithm identified 546 genes as most disruptive to the transcriptome correlations and for the 24 h samples 127 genes were identified as being de-phasing genes. The overlap between the dephasing sets was small (23 genes), but the genes identified as dephasing at 6 h showed consistent regulatory changes at 24 h (Additional file 2: S2–S5). The DI algorithm did not identify any genes as major disruptors in either the 38 h or 48 h samples. The shift in the 38 and 48 h curves results because the DI algorithm can make correlation curves arbitrarily precise, however the genes removed did not qualify as dephasing because the mutant and wild-type samples were already better correlated with each other than with the Derisi reference set

To identify the genes most prominently linked to the disrupted pattern of normal transcription, we developed, what is to our knowledge, a novel computational method to parse out the important differences between the datasets with a temporal sequence called the Dephaser Identifier (DI) algorithm. First, small numbers of genes were removed based on their absolute rank difference in expression between the wild-type and mutant strains and the correlations between the mutant and wild-type strain were recalculated. Subsequently, the process is performed iteratively until the mutant and wild-type strains have a correlation coefficient at least as high as the initial highest correlation of either the mutant or wild-type strain to the Derisi 3D7 reference transcriptome (Fig. 3).

**Fig. 4:**
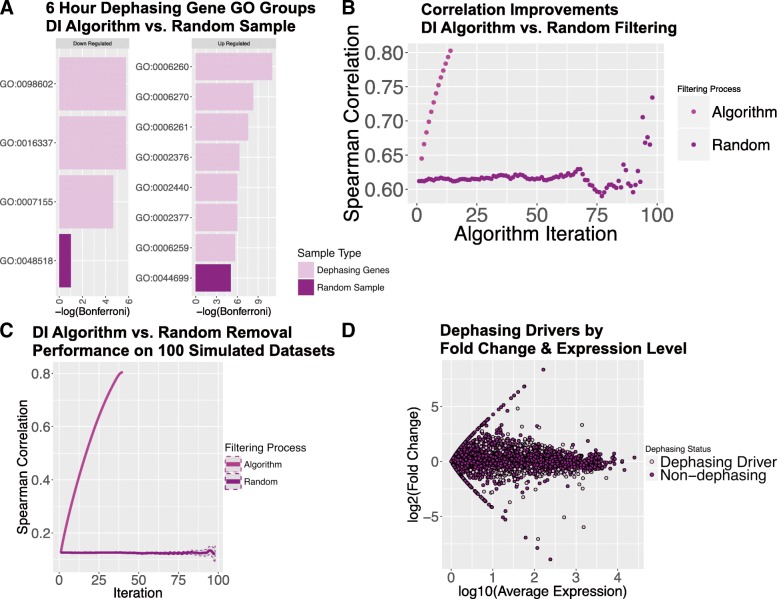
DI Algorithm performance. (**a**) The DI algorithm identifies more functionally related groups of genes than a random sampling of genes. Since functionally related genes tend to be regulated together this suggests that the DI algorithm is identifying genes that are differentially regulated between the wild-type and K13 mutant strains. At 6 h red blood cell invasion and exported proteins are over-represented amongst the down-regulated dephasing genes (GO:0098602--single organism cell adhesion, GO:0016337--single organismal cell-cell adhesion, GO:0007155--cell adhesion, GO:0048518--positive regulation of biological process,) and DNA replication and immune system processes are over-represented amongst the up-regulated dephasing genes (GO:0006260--DNA replication, GO:0006270--DNA replication initiation, GO:0006261--DNA-dependent DNA replication, GO:0002376--immune system process, GO:0002440--production of molecular mediator of immune response, GO:0002377--immunoglobulin production, GO:0006259--DNA metabolic process, GO:0044699--single-organism process). The graph for 24 h is Additional file 2: S1. (**b**) The DI algorithm consistently improves the correlations between 2 samples with each iteration while randomly removing genes does not. (**c**) The DI algorithm was tested on 100 simulated data sets and consistently identified the genes responsible for the poor correlations between the samples while randomly removing genes did not. The grey areas around the curves are the 95% confidence intervals. (**d**) Filtering out lowly expressed genes prevents the DI algorithm from identifying high variance-low confidence genes as dephasing (Additional file 2: S7). This volcano plot shows that the identification of dephasing driver genes is not biased by expression level like other methods of detecting transcriptome differences between samples

Our computational procedure identified 546 genes primarily responsible for de-phasing of the rhythmic structure of the mutant 6-h IDC correlation curve. There are 305 genes that show an increase in their expression rank and 241 genes that show a decrease in their expression rank. Over-represented amongst the increased expression rank set are genes involved in DNA replication and DNA replication initiation (Bonferroni corrected *p*-values < 0.0005; Fig. 4a) and in the decreased expression rank set genes involved in host cell invasion are over-represented (Bonferroni corrected *p*-values < 0.005; Fig. 4a).

The genes identified as dephasing at 6 h show consistent changes in expression at 24 h (the time point where *k13* becomes aberrantly up-regulated). The increased expression rank de-phasing genes at 6 h are significantly down-regulated at 24 h and the decreased rank expression genes are significantly up-regulated at 24 h (*p*-values Determined by Wilcoxon Rank Sum test on fold changes and for more information on the statistics see Additional file 2: S2 and S3). For both increased and decreased expression rank 6-h dephasing genes there are consistent shifts in relative rank with decreased rank expression dephasing genes having higher relative ranks in the mutant compared to the wild-type at 24 h and 6 h increased rank expression de-phasing genes are more likely to have lower relative rank compared to the mutant than expected by chance (*p*-values from Wilcoxon rank sum test between de-phasing genes and random samples. See Additional file 2: S4 and S5 for more information on the statistics). The statistical evidence is much stronger for the 6-h up-regulated dephasing genes; since *k13* is aberrantly down-regulated at 6 h and aberrantly up-regulated at 24 h together these results suggest K13 acts as a negative regulator of this de-phasing gene set.

The DI algorithm identifies more biologically consistent gene sets as dysregulated compared to randomly sampled genes (Fig. 4a). Of 100 dephasing gene sets created by randomly removing genes, only 3 (*p*-value = 0.03) created as many statistically significant gene sets as the DI algorithm (Additional file 2: S6). Furthermore, two independent control methods confirms that the DI algorithm produces highly specific results. For the first control method, we randomly removed genes from the transcriptome datasets, and we show that randomly removing genes does not change the transcriptome IDC correlations between 2 samples (Fig. 4b). For the second control method, we ran our DI algorithm on 100 simulated datasets, which showed that the DI algorithm consistently identifies the genes that decrease sample correlation each iteration whereas randomly removing genes does not (Fig. 4c). To increase confidence in our results, we removed lowly expressed genes before filtering to prevent the DI algorithm from being biased towards lowly expressed high variability genes with large fold changes (Fig. 4d; Additional file 2: S7).

### Analysis of differentially expressed gene sets

**Fig. 5:**
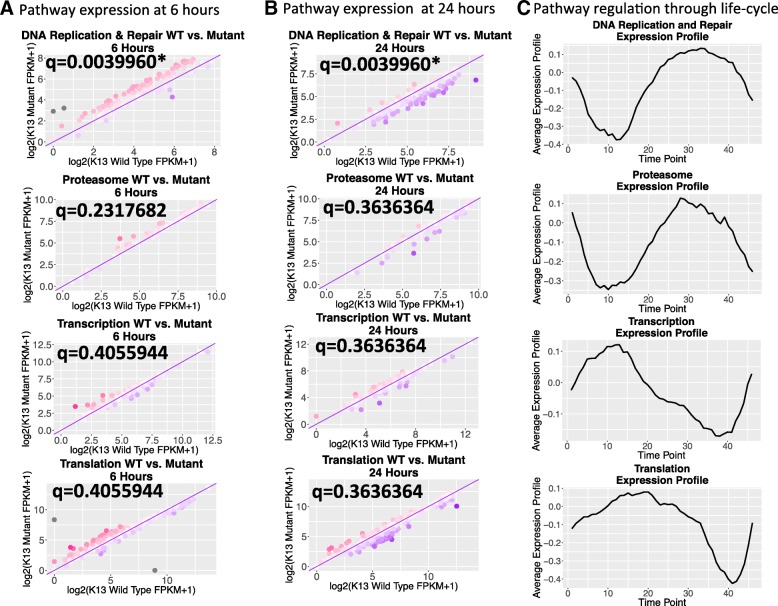
DNA replication and repair pathways are dysregulated, but housekeeping pathways are not. There are clear shifts in the expression patterns of the DNA replication and repair genes that are not apparent in other gene sets that also undergo rapid transcriptional regulation at the same points of the life-cycle. As indicated by the data from Bozdech et al. 2003, the proteasome, transcriptional machinery and translational machinery (Additional file 3) all undergo rapid changes in transcript expression levels around 6 and 24 h of the intraerythrocytic life-cycle (**c**) but these gene sets show consistent expression in the wild-type and mutant strains which supports the idea that the dysregulation observed in the DNA replication and repair genes is not due to time point sampling error but results from the dysregulation of *k13*. Grey dots represent absolute fold changes greater than 2.5. The DNA replication and repair pathways were combined into a single plot because they undergo equivalent rates of transcriptional regulation (Additional file 1: S8). The q statistic in (**a**) and (**b**) refers to the false discovery rate

To confirm that regulation of DNA replication is significantly disrupted in the mutant, gene set enrichment analysis using GAGE was performed on all *P. falciparum* pathways in KEGG [26, 27]. The enrichment analysis showed that DNA replication and repair is up-regulated in the mutant at 6 h and interestingly the same pathways are down-regulated in the mutant at 24 h, when K13 is up-regulated. This pathway analysis supports *k13* being a negative regulator of DNA replication and repair (Table 1). To confirm that DNA replication and repair alterations in expression are specifically dysregulated in the mutant transcriptome, we compared their expression changes to other housekeeping pathways (Additional file 3) that are also actively transcriptionally regulated around 6 and 24 h (Fig. 5; DNA replication and repair genes combined into one graph since they have similar expression profiles—Additional file 1: S8). Our results revealed DNA replication and repair pathways are specifically disrupted at 6 and 24 h, in contrast to housekeeping genes of the proteasome, transcription, and translation. These housekeeping genes have no shift in expression despite undergoing similar rates of transcriptional regulatory changes around the 6- and 24-h time points (Fig. 5c) [25]. Further, DNA replication and repair genes are normally expressed at higher levels at 24 h than at 6 h (Additional file 1: S9A) [25], which is similar in the wild-type strain NF54 (Additional file 1: S9B). However, for the *k13* mutant, DNA replication and repair genes actually have higher expression levels at 6 h then 24 h (Additional file 1: S9B). These data indicate that the regulatory effect of *k13* is specific for cell-cycle dependent differentially-regulated genes, but not for others. In particular, DNA replication and repair expression levels change consistently in response to differential *k13* expression, but not in the other housekeeping pathways analyzed. In this *k13 piggyBac* mutant parasite clone that is more sensitive to artemisinin, DNA replication and repair expression levels at 24-h actually fall below their 6-h expression levels when *k13* is up-regulated strongly. This result is interesting because in artemisinin-resistant strains resistant *k13* polymorphisms are associated with the down-regulation of DNA replication genes during the ring-stage [11] and in the trophozoite and schizont stages [28], indicating that regulation of DNA replication genes is linked to the artemisinin resistance response.

**Table 1:** KEGG pathways dysregulated in the mutant

6 hour up-regulated KEGG pathways						24 hour down-regulated KEGG pathways					
KEGG Gene set	p.geomean	p.stat.mean	p.val	q.val	set.size	KEGG Gene set	p.geomean	p.stat.mean	p.val	q.val	set.size
pfa03030 DNA replication	2.49E-05	4.50	1.70E-12	7.48E-11	29	pfa03030 DNA replication	5.67E-05	−4.21	2.02E-17	8.90E-16	29
pfa03430 Mismatch repair	2.83E-03	2.96	1.18E-06	2.60E-05	19	pfa03430 Mismatch repair	7.03E-03	−2.51	8.72E-08	1.92E-06	19
pfa03440 Homologous recombination	2.08E-02	2.17	2.61E-04	2.61E-03	13	pfa03440 Homologous recombination	2.61E-02	−1.86	6.82E-05	7.50E-04	16
pfa03410 Base excision repair	2.31E-02	2.05	4.57E-04	2.87E-03	16	pfa03410 Base excision repair	4.08E-02	−1.77	1.08E-04	9.48E-04	13

Definitions of terms: **p.geomean**: geometric mean of *p*-values from pairwise sample comparisons. **stat.mean**: Average Mann Whitney U test statistic from pairwise sample comparisons. **p.val**: *p*-value for the assumption of no change in pathway regulation. **q.value**: False discovery rate corrected *p*-values. **set.size**: number of genes in the KEGG Gene Set

**Fig. 6:**
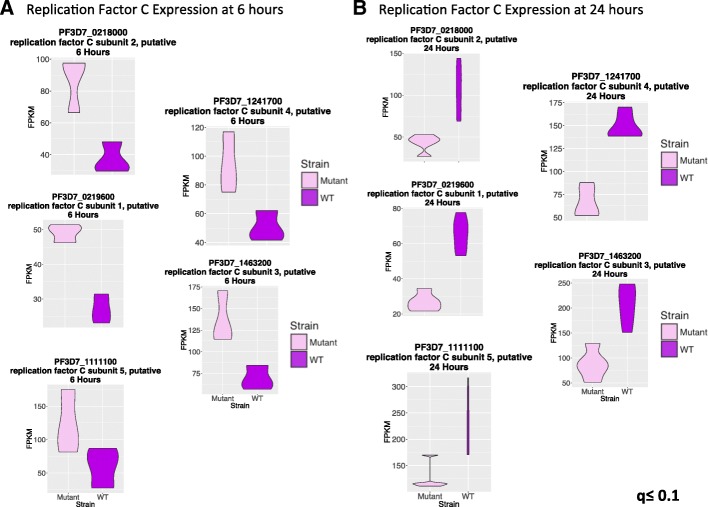
Replication factor C complex is dysregulated in K13 mutant. Network analysis links K13 to DNA replication in general and replication factor C in particular [29]. The replication factor C subunits show consistent changes in expression at 6 and 24 h (**a**, **b**) consistent with K13 being a negative regulator of DNA replication. Microarray measurements from theses time points are also consistent with the RNA-seq data presented here (Additional file 1: S10). The false discovery rate (q) was less than or equal to 0.1 for all comparisons made

Chemogenomic profiling of *P. falciparum* isogenic mutants has previously linked K13 to DNA replication and repair [21]. Further, the functional interaction network of *P. falciparum* [29] available as plasmoMap predicts that DNA replication linked genes are over-represented among the predicted functional interaction partners of K13 (Additional file 4—compiled predictions from 3D7, HB3, and Dd2 with a minimum threshold of 2.5). In particular, 4 of the 5 components of DNA replication factor C complex are present in the predicted K13 functional interactors with a fold enrichment of 8.02 and false discovery rate of 0.0007 as computed by PlasmoDB. Indeed, our RNA-seq and microarray results are consistent with this prediction (Fig. 6 and Additional file 1: S10) showing up-regulation of DNA replication factor C (PF3D7_0219600, PF3D7_0218000, PF3D7_1463200, PF3D7_1241700, PF3D7_1111100) at 6 h and down-regulation at 24 h.

**Fig. 7:**
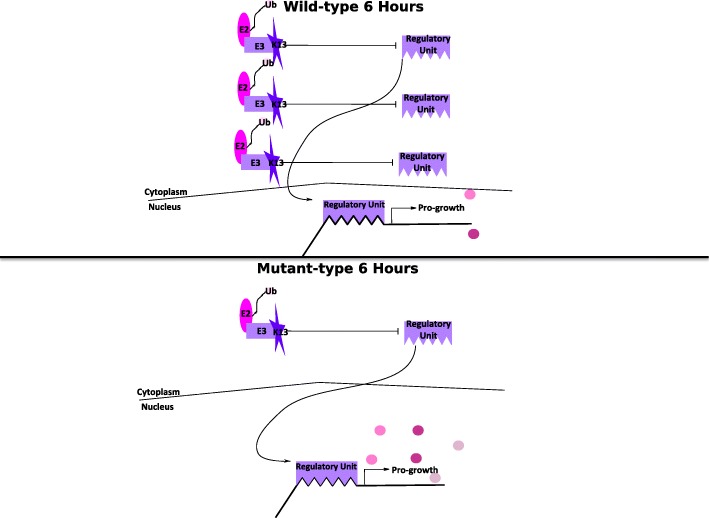
Proposed model of K13’s biological role. The crystal structure of K13 resembles the E3 ubiquitin substrate ligase adapter Keap1, which is known as a stress response regulator in humans. The structural similarity and the data suggest that K13 functions similarly to Keap1 promoting the inhibition of a pro-growth regulatory unit via ubiquitination of a regulatory element that is subsequently degraded by the proteasome. In this model down-regulation of K13 at 6 h would result in an increased number of functional regulatory units promoting the transcription of pro-growth genes, which would explain the transcriptome shift at 6 h towards latter life-cycle transcriptomes

The structural similarity of K13’s BTB and propeller regions to human Keap1, which is a known negative regulator of transcription [18], supports the functional interaction observed in *P. falciparum* between K13 and the DNA replication and repair genes and likely results from K13 regulation of a malaria parasite transcription factor. To identify the most likely regulated transcription factor, we looked for over-representation of dysregulated genes among genes with promoter sequences associated with transcription factors that regulate DNA replication genes described in Campbell et al. [30], using Fisher’s exact test (Additional file 5). In this analysis, we found that the 6-h increased rank de-phasing genes disproportionally (Bonferroni adjusted *p*-value 7.2e-6) have a promoter binding site for AP2 domain transcription factor, putative (PF3D7_0802100). This suggests that this AP2 domain transcription factor is a positive regulator of growth negatively regulated by K13 via ubiquitination (Fig. 7).

## Discussion

Detecting differentially-expressed genes is notoriously difficult in *P. falciparum* [31, 32] and this problem is evidenced here by dramatic alterations to the transcriptome that were undetectable at the individual gene level. In this study, the difficulty of identifying differentially-expressed genes stemmed from the low average fold-change of only 1.4 for the most dysregulated genes. However, small but coordinated changes in pathway gene expression can have large phenotypic effects [33] and the DI algorithm revealed that small but consistent changes were occurring in genes with common biological processes consistent with the identified dysregulated genes being linked by co-regulation. We statistically verified that dysregulation was occurring amongst the largest gene set (DNA replication) identified as dysregulated by the DI algorithm. Thus, the DI algorithm provides an unbiased way to identify sets of genes to be examined for changes in expression.

The *k13* mutant (PB58) in this study came to our attention through a chemogenomic screen of isogenic mutants that identified it as being more sensitive to artemisinin antimalarial drugs than the wild-type parent NF54 strain [21]. This mutant has a transposon in the 5′ upstream region of *k13*, suggesting dysregulation of *k13* expression led to an altered drug sensitivity phenotype, which this study confirmed. The fact that this dysregulation occurs at 2 different stages of the IDC and involves both up and down-regulation of *k13* allows the direct testing of the effect of K13 expression on the broader transcriptome.

DNA replication was identified as the biological process with the largest enrichment amongst the dephasing genes. Subsequently, we confirmed that DNA replication and repair pathways are the most dysregulated of the *P. falciparum* pathways annotated in KEGG. The dysregulation of DNA replication and repair genes is specific as evidenced by the fact that other housekeeping pathways that undergo similar rates of transcriptional regulation show no shifts in their expression levels. The unique *k13* expression profile of this mutant provides further evidence for this phenotype with down-regulation of K13 corresponding to an up-regulation of DNA replication and repair and up-regulation of K13 corresponding to a down-regulation of DNA replication and repair.

*k13* is the gene with the strongest observed link to the artemisinin resistance phenotype observed with the in vitro ring survival assay [34]. The link to artemisinin’s mechanism of action is evident in the isogenic *k13* mutant studied here because it is more sensitive to arteminsin drugs than its wild-type parent strain NF54. These previous data together with the transcriptome alterations revealed by our analysis using the DI algorithm suggests that K13 functional changes are relevant to *P. falciparum’s* response to artemisinin. Other studies [17, 35] indicate that the resistance associated *k13* alleles have decreased target binding; however, the increased susceptibility of this *k13* mutant to arteminsins is puzzling because K13 is down-regulated during the early ring stage. A logical conclusion of this observation is that the increased sensitivity would occur during the early trophozoite stage during which *k13* transcript levels are likely either up-regulated due to the calmodulin promoter or could participate in a negative feedback loop to suppress the premature pro-growth phenotype. K13’s homology to Keap1 and the regulation of DNA replication and repair as detected here are consistent with K13 being a stress response regulator. A role for K13 regulating DNA replication and repair comports with previous studies that found artemisinin resistant strains down-regulate DNA replication genes [11, 28] and previous network analysis studies that linked K13 to DNA replication and repair [21, 29].

## Conclusion

Understanding K13’s function is important to understand the mechanism of artemisinin resistance. Given that K13 is likely essential for parasite survival, regulatory mutants are one of the important ways to study K13. This work compared the transcriptional profiles of isogenic strain pairs of *P. falciparum* with divergent K13 regulation during the IDC. The points of dysregulation show consistent and specific disruption to the normal expression patterns of DNA replication and repair genes. This finding supports the proposed function of *k13* as a regulator of stress response based on *k13*’s homology to *KEAP1* and is consistent with previous network analysis studies that linked *k13* to DNA replication and repair [21, 29] and showed that artemisinin resistant strains down-regulate DNA replication genes [11, 28].

## Methods

### Parasite culture and sequencing

#### RNA -seq

The parasite strains NF54 and PB58 (the K13 mutant) [21] were maintained in identical standard culture conditions and synchronized by 3 rounds of sorbitol synchronization. The time points collected were 6 (*n* = 3), 12 (*n* = 2), 24 (*n* = 5), 38 (*n* = 3), and 48 (*n* = 3 for wild-type and *n* = 2 for K13 mutant) hours after time zero. Time zero was defined as the time when the synchronized culture was half late schizonts and half early rings. When a culture reached a harvest time point the parasites were separated from the red blood cells with 0.015% saponin at room temperature for 5 min. The parasites were then pelleted and washed three times in 10 mL room temperature PBS and the samples were stored at − 80 °C in 1 mL TRIzol reagent (Fisher Scientific, Hampton, NH) until extraction. For extraction 200 μl of chloroform was added and the samples vortexed vigorously for 15 s and then incubated at room temperature for up to 5 min. The samples were then spun down at 12000×g (10,800 rpm) at 4 °C for 10 min and the supernatant discarded. 1 mL of 75% ethanol was added and then the samples spun down at 10000×g (9800 rpm) for 5 min. The resulting supernatant was discarded and the pellet briefly allowed to dry and the pellet dissolved in 20–50 μl of DEPC-treated water while being incubated at 55 °C for 10–15 min.

0.5 μg–1.0 μg of RNA samples were prepped for sequencing using the Illumina TruSeq Stranded mRNA Kit. Library quantification was measured by qPCR and TapeStation (Agilent Technologies). Sequencing was performed on an Illumina MiSeq using 300-cycle V2 MiSeq reagent kit (Illumina).

#### Microarray

The microarray measurements were performed as described in [36]. Briefly, RNA was extracted using TriZol reagent (Invitrogen, Carlsbad, CA) and the quality and quantity determined by NanoDrop (NanoDrop Technologies). 300 ηg of RNA was used for cDNA synthesis using Sigma WTA2 whole transcriptome amplification kit. 1 μg of cDNA was labeled with Cy3 dye and allowed to hybridize to a custom Agilent array for 17 h followed by washing. The microarray image was taken using a 2 μM scanner and probe intensity values obtained using Agilent Feature Extraction software. Normalization of probe intensities was done using the robust multichip average (RMA) method. The time points were obtained as described above for the RNA-seq measurements and include the time points 6 (*n* = 3), 24 (*n* = 3) and 38 (*n* = 3) hours after time zero.

Ethical approval for the use of human blood in this study was granted by the Institutional Review Boards of the University of South Florida and the University of Notre Dame. All of the blood used for the in vitro culturing of parasites was obtained from healthy adult volunteers and drawn by trained personal from Interstate Blood Bank.

The NF54 strain was originally obtained from the Naval Medical Research Center.

### Obtaining gene expression data

Reads were aligned to 3D7 reference release 27 using HISAT2 version 2.0.4 [37]. Raw counts were obtained using FeatureCounts Version 1.50.0-p3 [38] . Transcripts were assembled using Cufflinks Version 2.2.1 and FPKM (Fragments per kilobase per million mapped reads) values calculated using Cuffnorm Version 2.2.1 using the classic-fpkm setting and normalization was performed by strain and time point [39]. Expression data available as Additional file 6.

Since lowly expressed genes are more subject to stochastic fluctuations mitochondrial and apicoplast genes as well as genes with less than 3 reads for every million reads sequenced in more than half the samples were removed from further consideration.

### Identification of sample outliers

TMM (trimmed mean of M-values) normalized count data was used to calculate Pearson correlation pairwise between all replicates. If a sample had a correlation of less than 0.7 with at least 2 other replicates it was removed as an outlier. This cutoff was chosen based on the fact that most of the biological replicates had correlation coefficients of at least 0.7, but a few had correlation coefficients that were lower.

### Determination of K13 dysregulation

To test differential expression of *k13* at 6 and 24 h the wilcox.test in R version 3.4.1 was used to implement the nonparametric Mann-Whitney test. The input was the FPKM values for *k13* and the samples were tested for down-regulation of K13 at 6 h and up-regulation of K13 at 24 h. The Holm procedure in R version 3.4.1 was used to adjust *p*-values for multiple testing [40].

### EdgeR analysis

Differential expression analysis was performed using EdgeR version 3.18.1 [41]. As previously noted the counts used as input to EdgeR were obtained using FeatureCounts Version 1.50.0-p3. Mitochondrial, apicoplast and genes with less than 3 counts per million in more than half the samples were not considered (filtered as previously described). TMM (trimmed mean of M-values) normalization [42] was performed prior to differential expression analysis and differential expression was tested between strains at each time point.

### Determination of DNA replication factor C dysregulation

The differential expression of the DNA replication Factor C components was performed following the same procedure described under “Determination of K13 Dysregulation” for the 6 and 24 h time points except the false discovery rate was used to adjust *p*-values [43].

### Gene set analysis

All *P. falciparum* pathways annotated in KEGG on September 4 2017 were analyzed for differential expression using Gage 2.26.3 via the Mann Whitney U test on unpaired samples [26].

GSAR version 1.10.0 was used to perform a KStest [44] on DNA replication and repair, proteasome, transcription and translation gene ontology sets obtained from PlasmoDB [45]. The lists of genes used to form the gene sets is found in Additional file 3.

To see if the down-regulated dephasing genes at 6 h are more likely to be up-regulated at 24 h we checked to see if the fold-changes of these genes are higher than the fold changes of a random sample of genes. The same is done for the up-regulated dephasing genes, but now they are expected to be more down-regulated. More specifically, a Wilcoxon rank sum test on the log2 fold changes of the FPKM values between the mutant and wild-type strains at 24 h was performed and the results compared to random samples. For the up-regulated dephasing genes the controls were genes that also showed an increase in relative rank at 6 h (*n* = 1704) and for the down-regulated dephasing genes the controls were genes that showed a decrease in relative rank at 6 h (*n* = 1951). This was performed 1000 times on different random samples to get the *p*-value distribution (Additional file 2: S3). The number of genes in each control set was equal to the number of genes in the experimental set.

A similar procedure to that described above was used to verify that the dephasing genes experienced consistent changes to their relative ranks at 24 h. For this test the input to the Wilcoxon rank sum test was the differences in the gene expression relative rank of the mutant and wild-type at 24 h. The resulting *p*-value distributions are shown in Additional file 2: S5.

### Mutant vs. wild-type similarity assessment

The sample transcriptomes were correlated with the Derisi 3D7 transcriptome [25] downloaded from PlasmoDB [45]. The steps to calculate the correlations are as follows:

1. Replicate FPKM values were averaged
2. The average value for a gene at a specific time point was divided by the average expression of that gene for all time points and samples and the log2 taken
3. The Spearman correlation between each sample time point was calculated with respect to each of the 3D7 reference IDC time points individually
4. Time point and strain clustering as well as heatmap creation were performed using the heatmap.2 function in gplots version 3.0.1

### Computational procedure to identify dephasing genes

Significant distortions to the mutant 6 h transcriptome were identified by plotting out line graphs of the Spearman correlation with the 3D7 reference transcriptome (calculated as described above). Given that very few differentially expressed genes were detected at this time point (Additional file 1: S7) we developed an algorithm named Dephaser Identifier (DI) detailed below to identify the genes responsible for the distortions to the IDC correlation curve. Prior to performing the procedure mitochondrial and apicoplast genes as well as genes with counts per million less than 3 in more than half the samples were removed and only genes present in our data set and in the Derisi reference transcriptome set were used. The DI algorithm is as follows:

1. Calculate relative gene expression level vectors for the control and mutant strains separately as follows

$$ {\log}_2\left(\frac{Average\ gene\ expression\  at\ a\  specific\ time\ point}{Average\ gene\ expression\ across\  all\  time\ points}\right) $$

2. Define a minimum acceptable correlation between the control and mutant strains. For this experiment the minimum acceptable correlation was defined as the highest spearman correlation that either the control or mutant strain had with a specific time point from the Derisi reference IDC time points [25] with the logic that 2 samples from the same time point and the same lab should correlate at least as well with each other as with a sample from a different lab
3. Rank the relative gene expression levels for both the control and mutant strains
4. For a given pair of time points and for each gene calculate the difference in rank between the control and mutant strains
5. Calculate the absolute value of the difference in rank for each gene
6. Assign the absolute value of the rank differences for each gene to quantiles (1% quantiles were used and the quantiles were calculated using the type 7 procedure in R version 3.4.1)
7. Remove the highest unfiltered quantile of genes from the relative gene expression level vectors for the control and mutant strains and calculate the Spearman correlation
8. Repeat step 7 until the Spearman correlation between the control and mutant strains is higher than the minimum acceptable correlation or there are no more genes left to filter

### DI algorithm performance assessment

To determine if the DI algorithm was identifying functionally related genes better than chance a simulation was run 100 times were a set of genes equal to the number of 6 h dephasing genes (*n* = 546) was randomly chosen and checked for biological process enrichment using topGO [46]. The minimum gene ontology set size (the node_size parameter in topGO’s run_enrichment_tests function) was set to 10 and a classic Fisher test was performed. Gene ontology terms with *p*-values of less than or equal to 0.05 were considered significant. The Bioconductor library org.Pf.plasmodb [47] was used to obtain the gene ontology annotations. The same procedure was performed once on the real 6 h dephasing gene set and the results compared to the simulation (Additional file 2: S6).

### Identification of transcription factor regulators

Genes with promoter regions linked to DNA replication regulating transcription factors were identified from the data set reported by Campbell et al. [30]. Campbell et al. linked 5 AP2 domain containing genes to DNA replication genes. The genes associated with each of these transcription factors were downloaded from PlasmoDB [45] if the *p*-value for an association with one of the transcription factors was less than or equal to 1 × 10^− 4^. As shown in Additional file 5 the genes associated with each of the transcription factors were used to partition the genome into genes predicted to have a binding site for the given transcription factor or not and whether or not the gene was identified as being an up-regulated 6 h dephasing gene or not to create contingency tables. The contingency tables were used to perform Fisher’s Exact Test in R version 3.4.1 and the *p*-values corrected using the Bonferroni method.

## Additional files

Additional file 1: Supplemental data pertaining to the K13 mutant and *P. falciparum* transcriptome. (PDF 1197 kb)

Additional file 2: Supplemental data pertaining to DI algorithm. (PDF 483 kb)

Additional file 3: Housekeeping gene sets. Genes sets for DNA replication and repair, translation, transcription and proteasome. (XLSX 70 kb)

Additional file 4: Predicted K13 functional interactors. Genes predicted to have functional interactions with K13 from plasmoMAP. (XLSX 57 kb)

Additional file 5: Fisher’s exact test results for transcription factor binding sites linked to dephasing genes. (XLSX 42 kb)

Additional file 6: Expression data. Expression data as counts and FPKM. (XLSX 2029 kb)

## Abbreviations

ACT: Artemisinin-based combination therapy
DI: Dephaser Identifier
FPKM: Fragments per kilobase per million mapped reads
GEO: Gene Expression Omnibus
IDC: Intra-erythrocytic development cycle
