# Supplementary material for: Altered expression of K13 disrupts DNA replication and repair in Plasmodium falciparum
Source: BMC Genomics. 2018 Nov 29;19:849. doi: 10.1186/s12864-018-5207-7 (PMC6263542; doi:10.1186/s12864-018-5207-7)
Supplement: Supplementary file 1 — Supplemental data pertaining to the K13 mutant and P. falciparum transcriptome. (PDF 1197 kb) [file 12864_2018_5207_MOESM1_ESM.pdf]

S1

K13 Piggybac Insertion Site

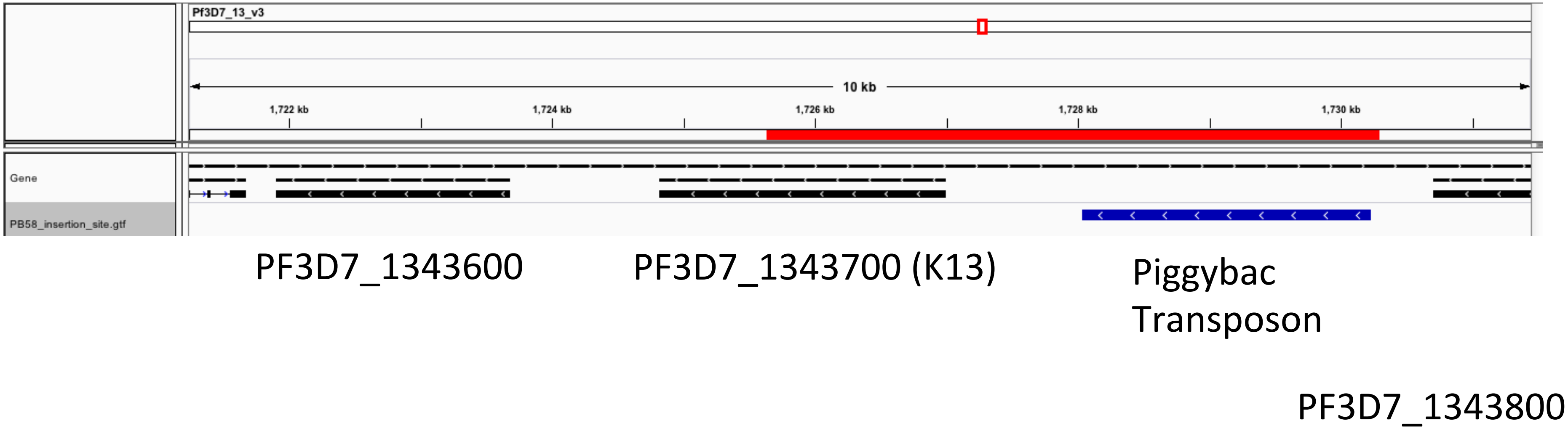

K13 mutant piggybac insertion site:
Location of the K13 mutant piggybac transposon 1034 nucleotides up-stream of the K13 gene (5' region is shown on the right-hand side because K13 is found on the negative strand).

S2

K13 Mutant Artemisinin Sensitivity

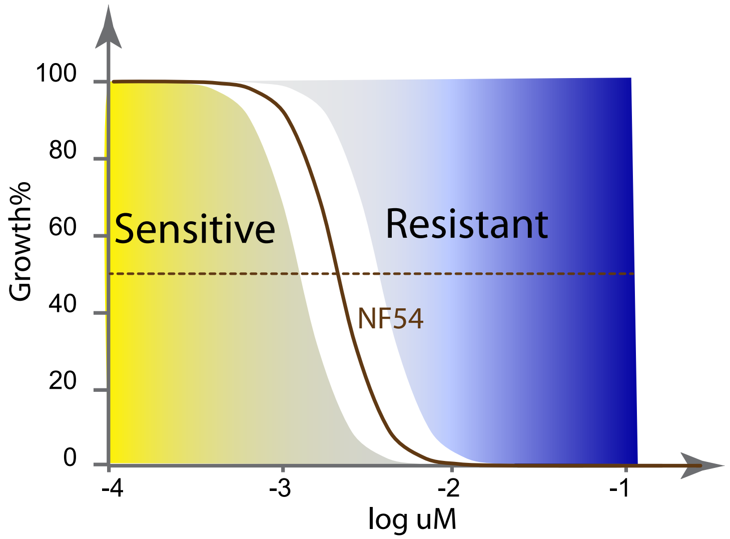

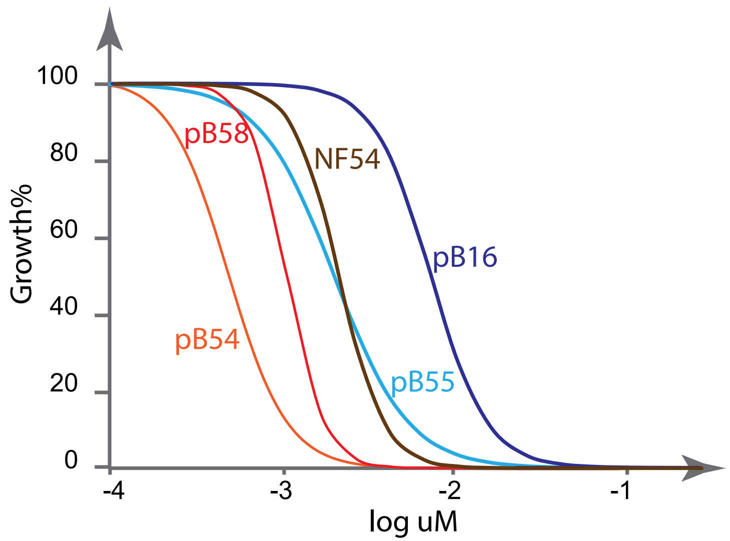

Pradhan et al 2015

GI_{50}'s of some piggybac mutants, including the K13 mutant (pb58).

S3

Microarray Validation of RNA-seq Results

6 Hour Up-regulated Pathways

gene_set	q-value
pfa03440 Homologous recombination	0.03988932
pfa03430 Mismatch repair	0.03988932
pfa03030 DNA replication	0.03988932
pfa03410 Base excision repair	0.07556977
pfa03420 Nucleotide excision repair	0.0896994

24 Hour Down-regulated Pathways

gene_set	q-vaule
pfa03030 DNA replication	4.90E-08
pfa03430 Mismatch repair	8.78E-05
pfa03410 Base excision repair	2.73E-04
pfa03420 Nucleotide excision repair	4.32E-03
pfa03440 Homologous recombination	4.32E-03

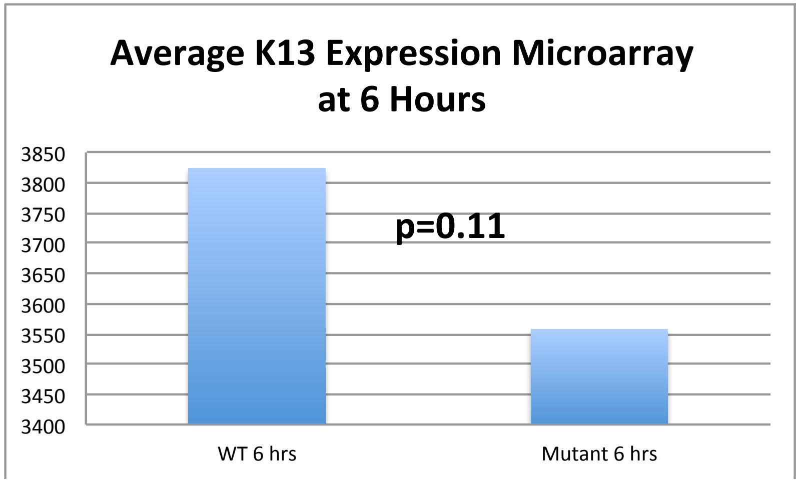

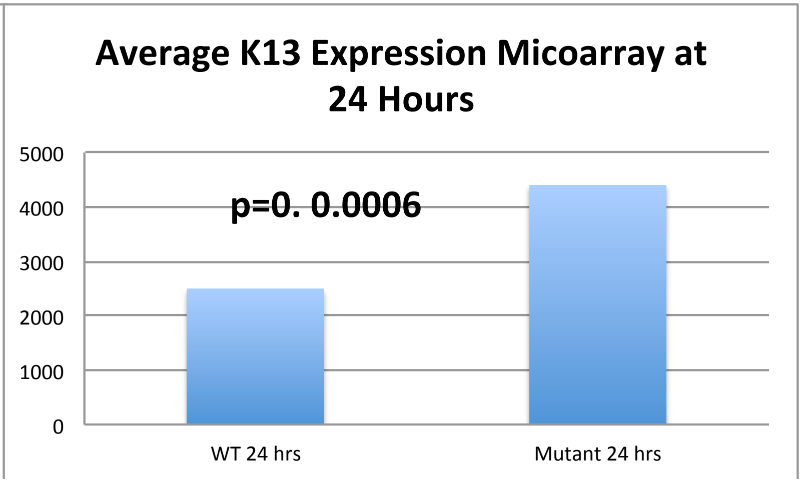

Microarray validation of RNA-seq results:
Microarray measurements are consistent with K13 being down-regulated in the mutant at 6 hours and up-regulated in the mutant at 24 hours.

S4

Sequencing Quality is Equivalent at 6 Hours Between the Mutant and Wild-type at 6 Hours

WT A

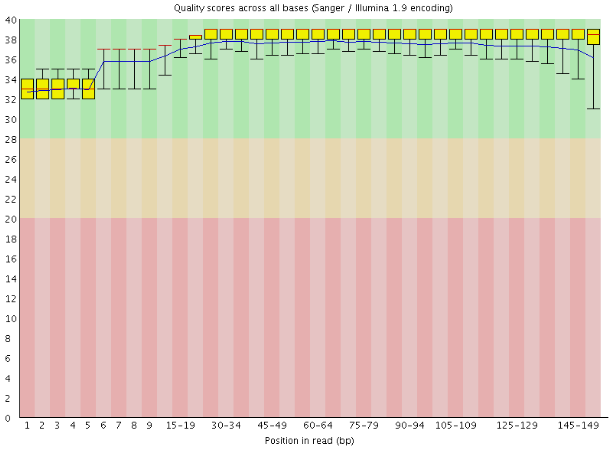

WT B

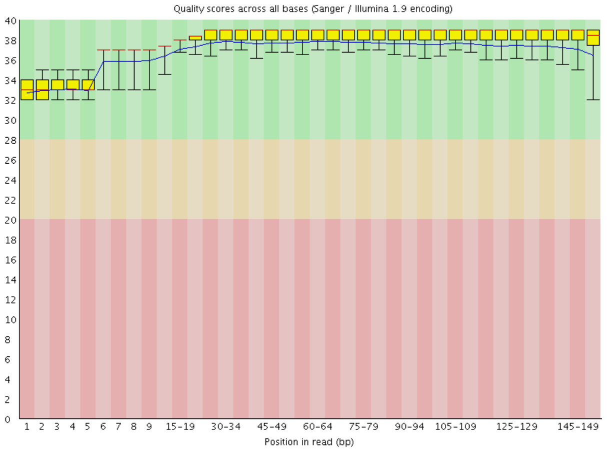

WT C

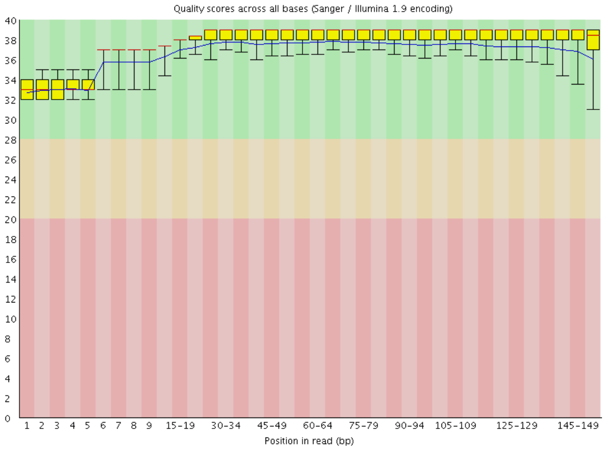

Mutant A

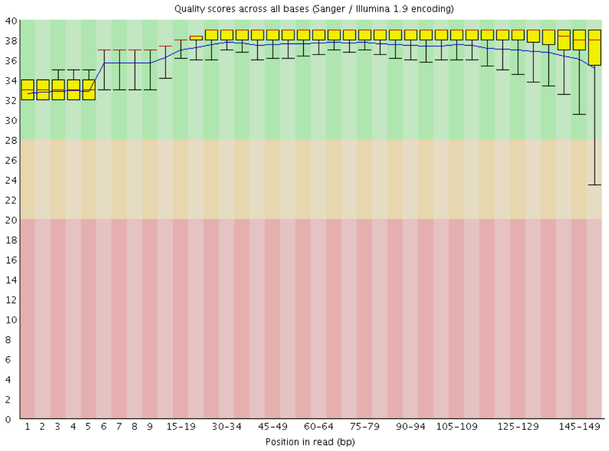

Mutant B

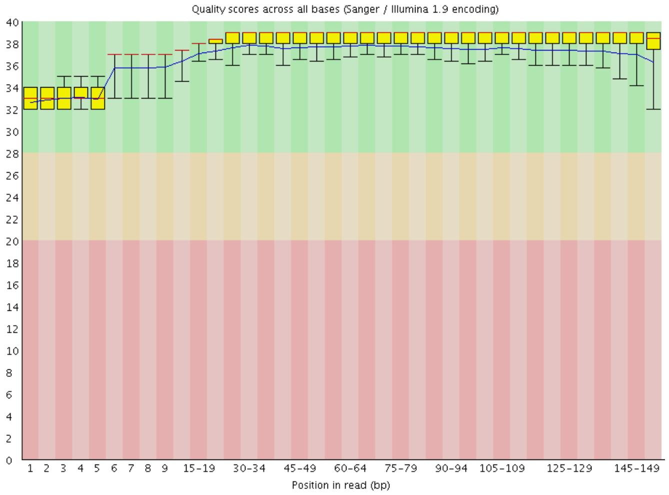

Mutant C

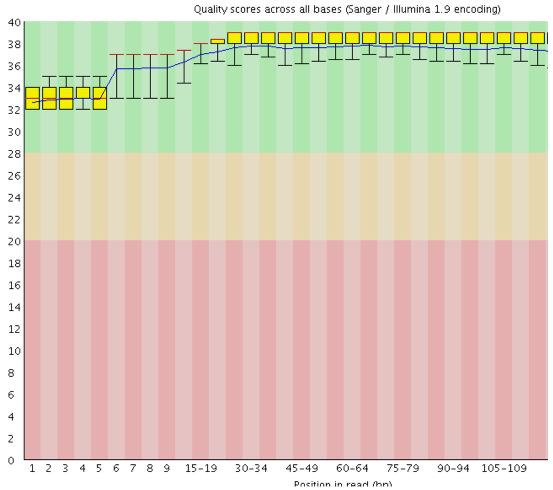

S9A. Average Expression profile for DNA replication and repair genes from 3D7 Strain

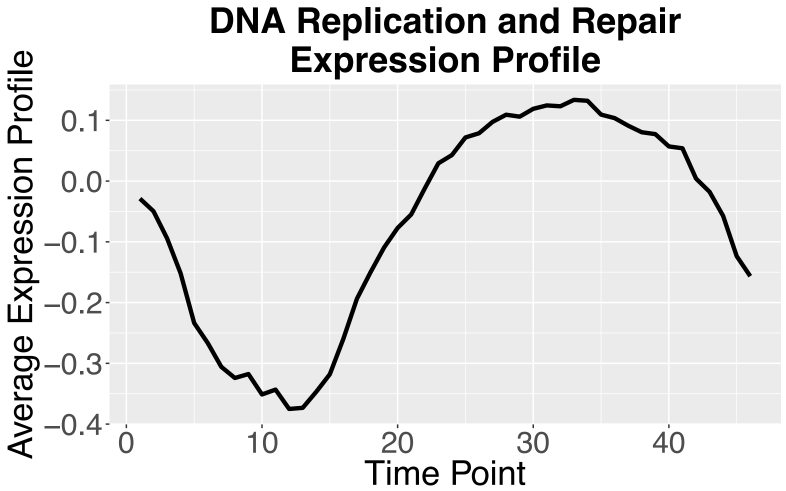

S9B.

Replication and repair expression between timepoints within the wild-type and mutant strains

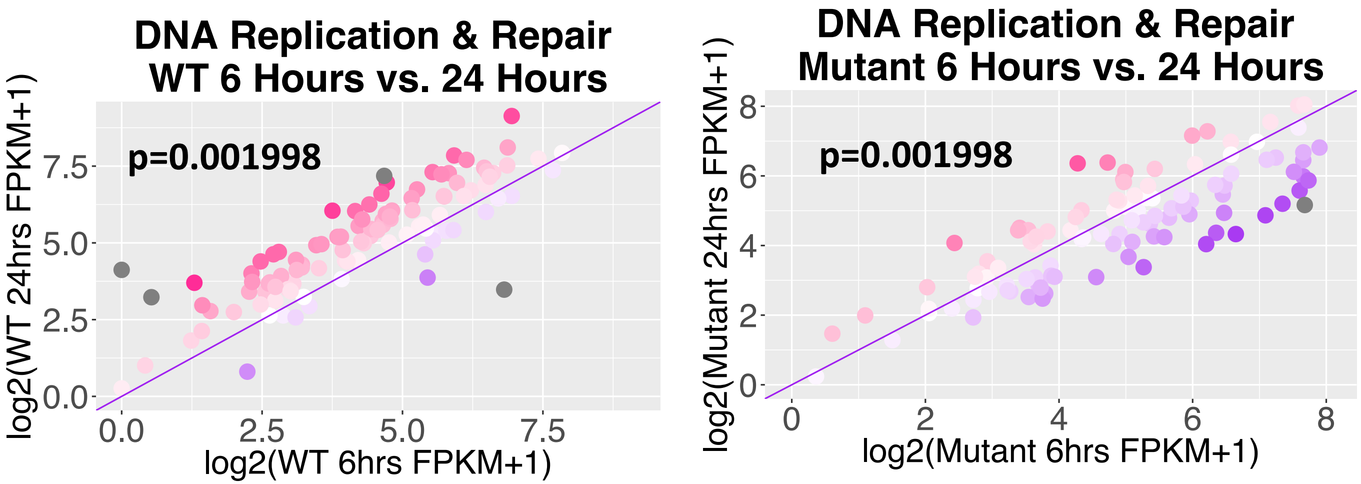

DNA replication and repair genes expressed at higher levels in mutant at 6 hours then 24 hours:
DNA replication and repair genes undergo down-regulation in the ring stage and become up-regulated during the transition to the trophozoite stage. Consequently DNA replication and repair genes normally have higher transcript levels at 24 hours then at 6 hours. (S9A) The normal pattern of higher DNA replication and repair transcript expression at 24 hours then 6 hours holds in the wild-type strain. (S9B) However, in the mutant DNA replication and repair genes are actually expressed at a higher level at 6 hours then 24 hours.

S5

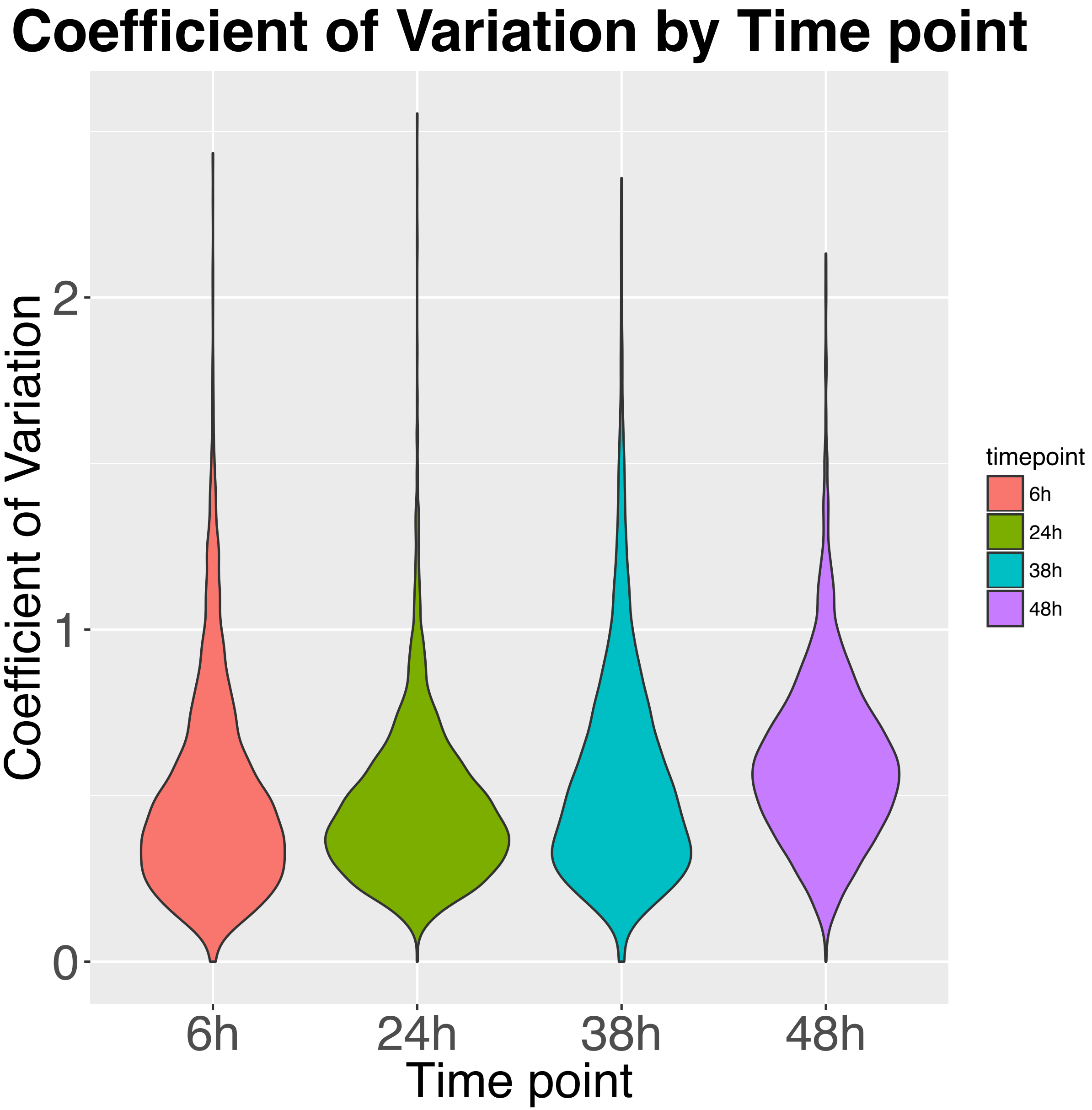

Sample variation by time point:
Variation within the 6 hour samples is no higher than the variation within the other time points meaning the observed transcriptome shift cannot be explained by increased sample variability.

S6

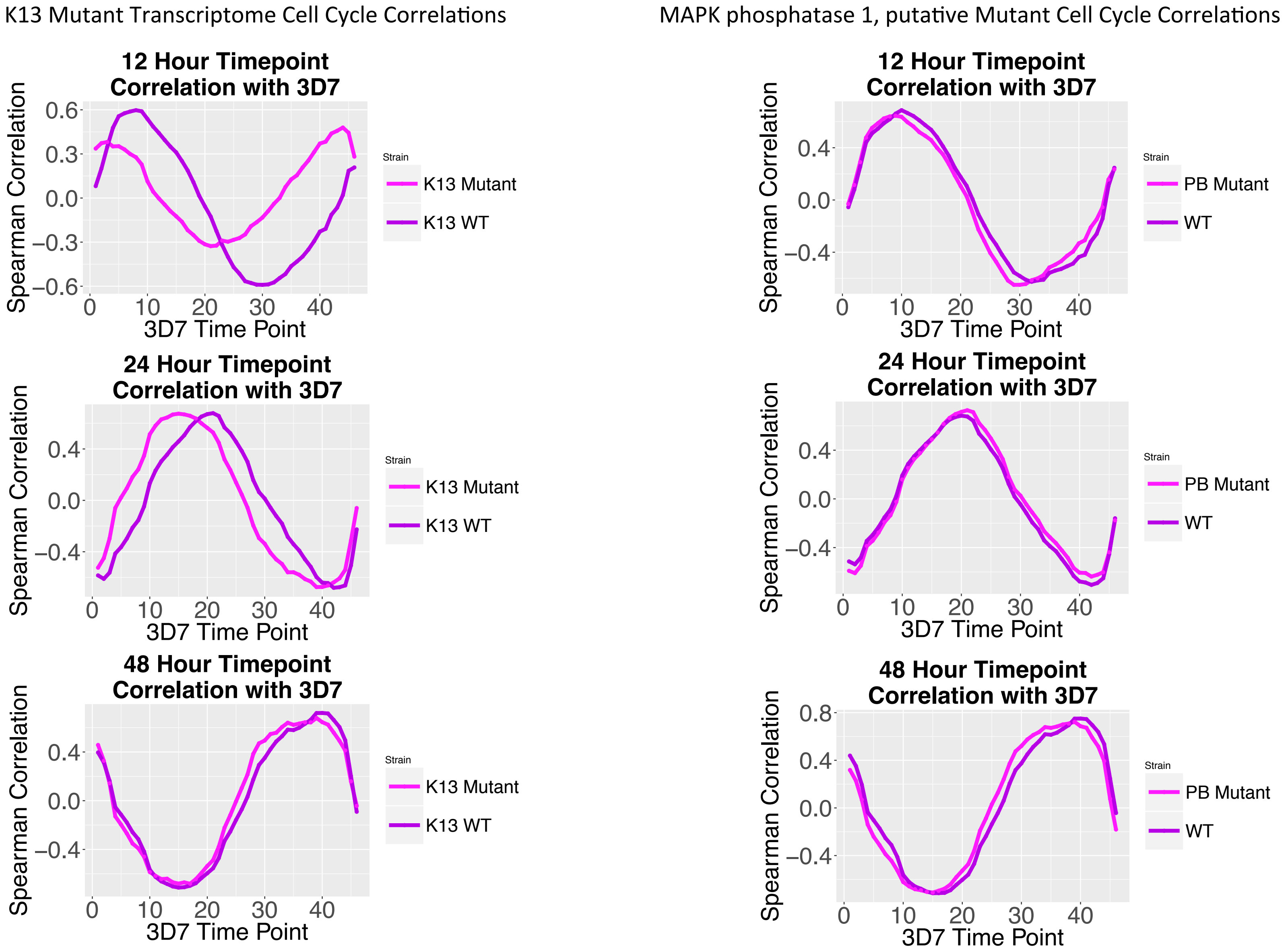

Transposon does not cause IDC shifts in other mutants:

The transcriptome of a piggyBac mutant (C9) with an insertion in the gene PF3D7_1305500 shows no IDC shifts relative to the parent strain suggesting that the transcriptomic shifts are not directly caused by the transposon. Note there is a cell cycle shift at 12 hours in the K13 mutant but this time point was not included in the main analysis because there were not enough samples to properly test shifts in pathway expression.

S7

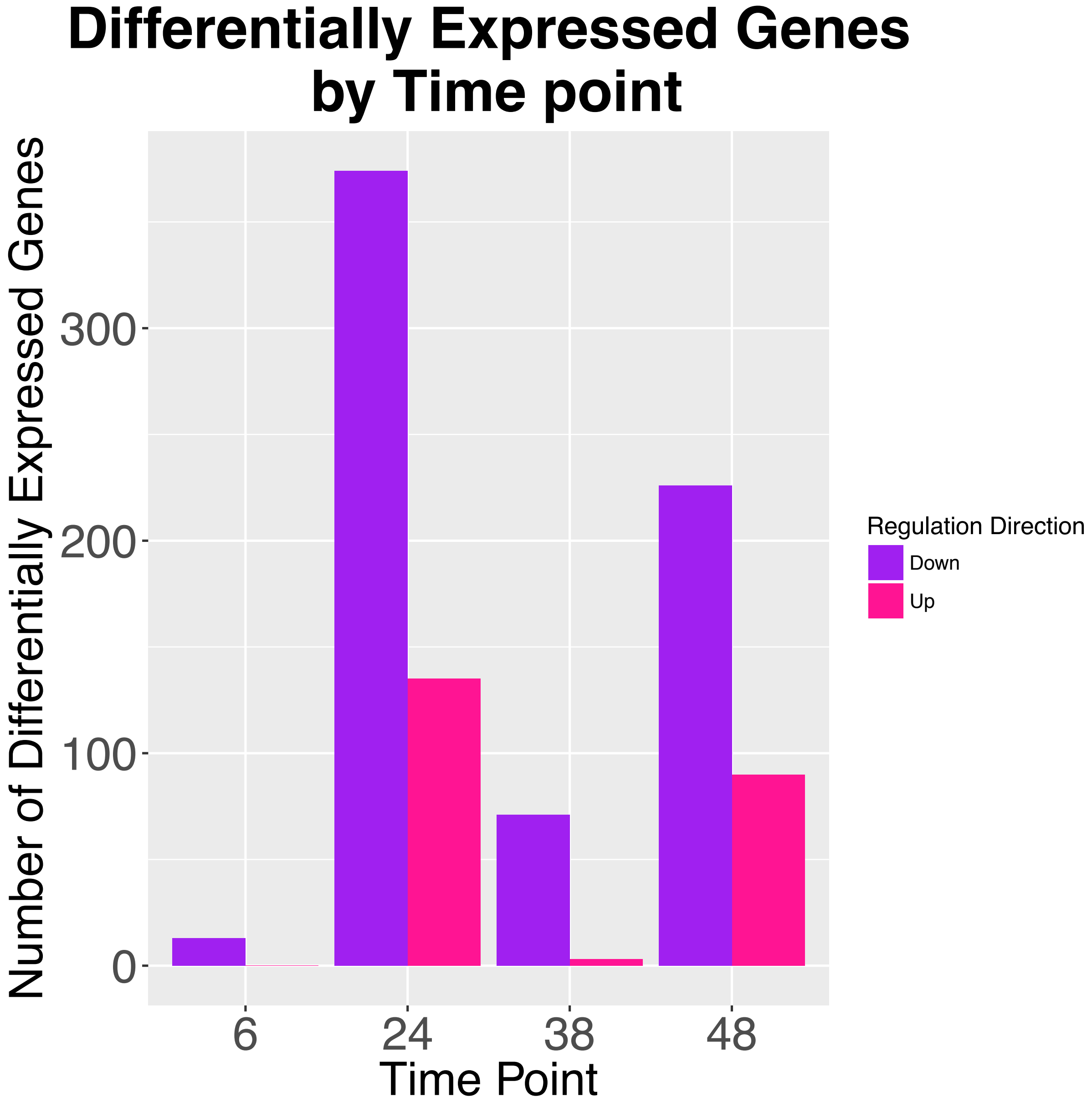

Individual gene analysis summary:
Differential gene expression analysis using EdgeR predicts that the 6 hour time point has the fewest number of differentially expressed genes which is puzzling since this is also the time point that displays the greatest divergence in the life-cycle correlation curve.

S8

DNA Replication and Repair Genes Have Similar Expression Profiles

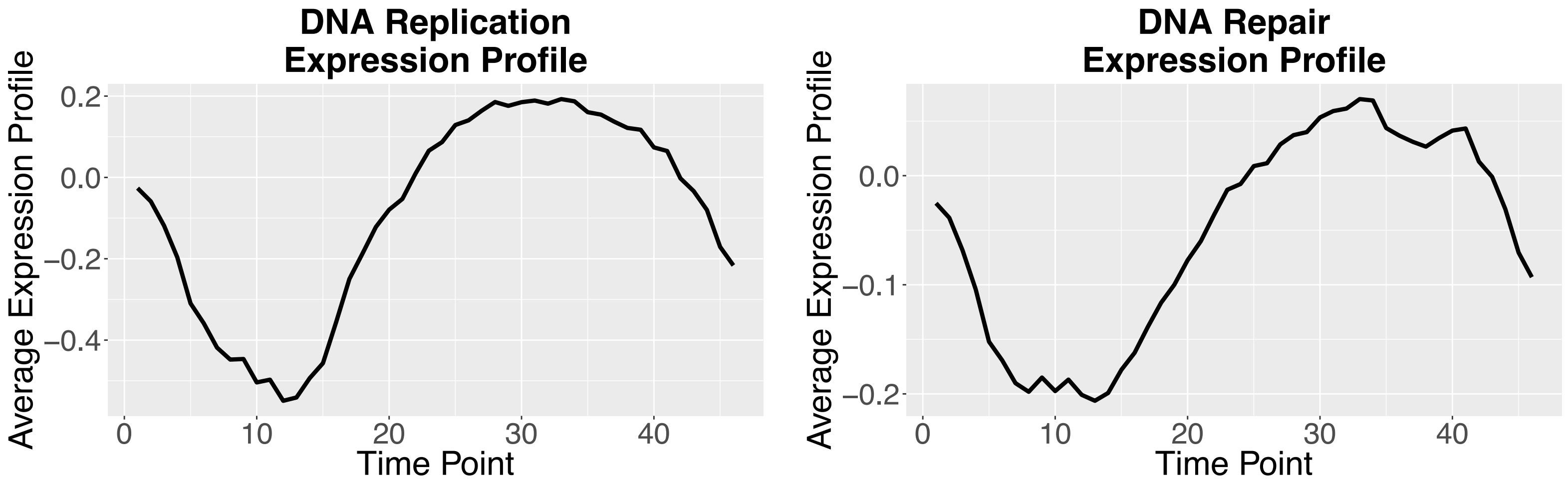

DNA replication and repair expression profiles:
The DNA replication and the DNA repair expression profiles have the same rates of transcriptional regulation throughout the cell cycle. This is the rationale for combining the DNA replication and repair genes for analysis.

S9A. Average Expression profile for DNA replication and repair genes from 3D7 Strain

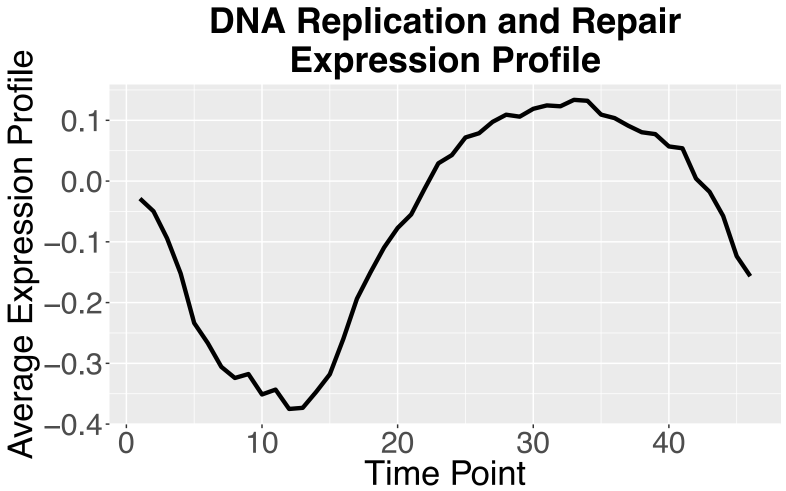

S9B.

Replication and repair expression between timepoints within the wild-type and mutant strains

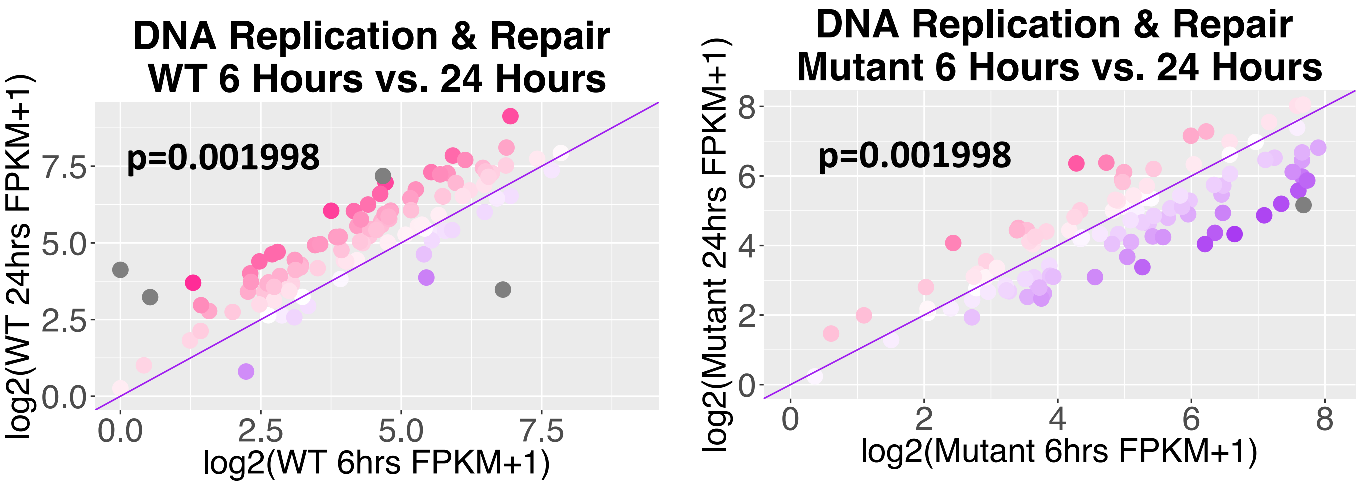

DNA replication and repair genes expressed at higher levels in mutant at 6 hours then 24 hours:
DNA replication and repair genes undergo down-regulation in the ring stage and become up-regulated during the transition to the trophozoite stage. Consequently DNA replication and repair genes normally have higher transcript levels at 24 hours then at 6 hours. (S9A) The normal pattern of higher DNA replication and repair transcript expression at 24 hours then 6 hours holds in the wild-type strain. (S9B) However, in the mutant DNA replication and repair genes are actually expressed at a higher level at 6 hours then 24 hours.

S10

Replication Factor C Expression by Microarray

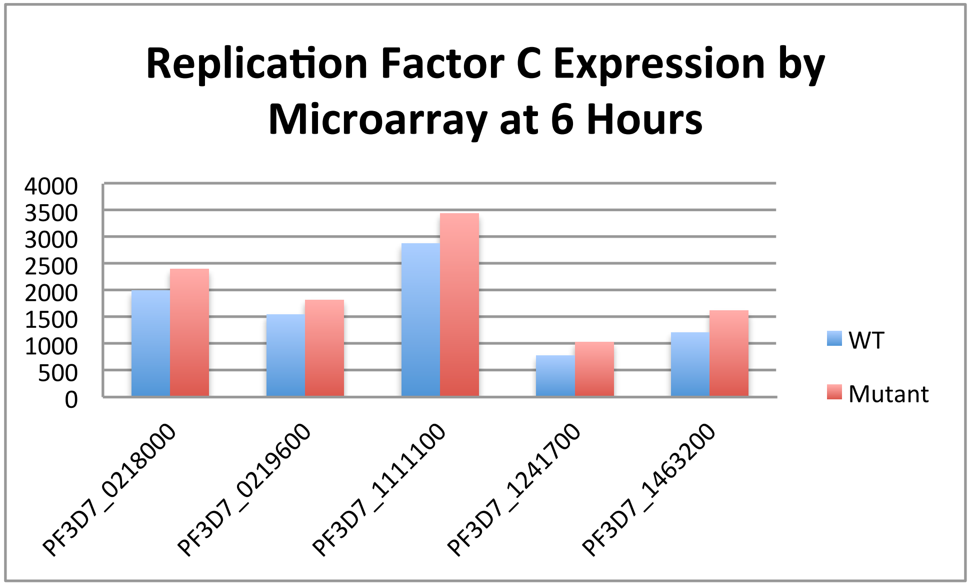

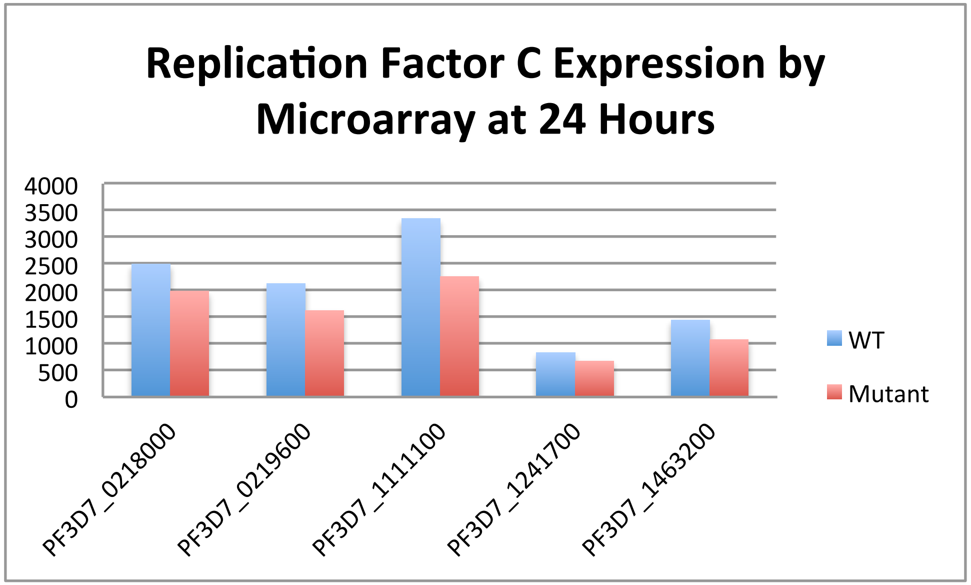

Microarray validation of Replication factor C complex dysregulation:
Microarray array measurements are consistent with RNA-seq measurements showing an up-regulation of the Replication factor C complex at 6 hours and down-regulation of the Replication factor C complex at 24 hours.
