# Supplementary material for: Altered expression of K13 disrupts DNA replication and repair in Plasmodium falciparum
Source: BMC Genomics. 2018 Nov 29;19:849. doi: 10.1186/s12864-018-5207-7 (PMC6263542; doi:10.1186/s12864-018-5207-7)
Supplement: Supplementary file 2 — Supplemental data pertaining to DI algorithm. (PDF 483 kb) [file 12864_2018_5207_MOESM2_ESM.pdf]

S1

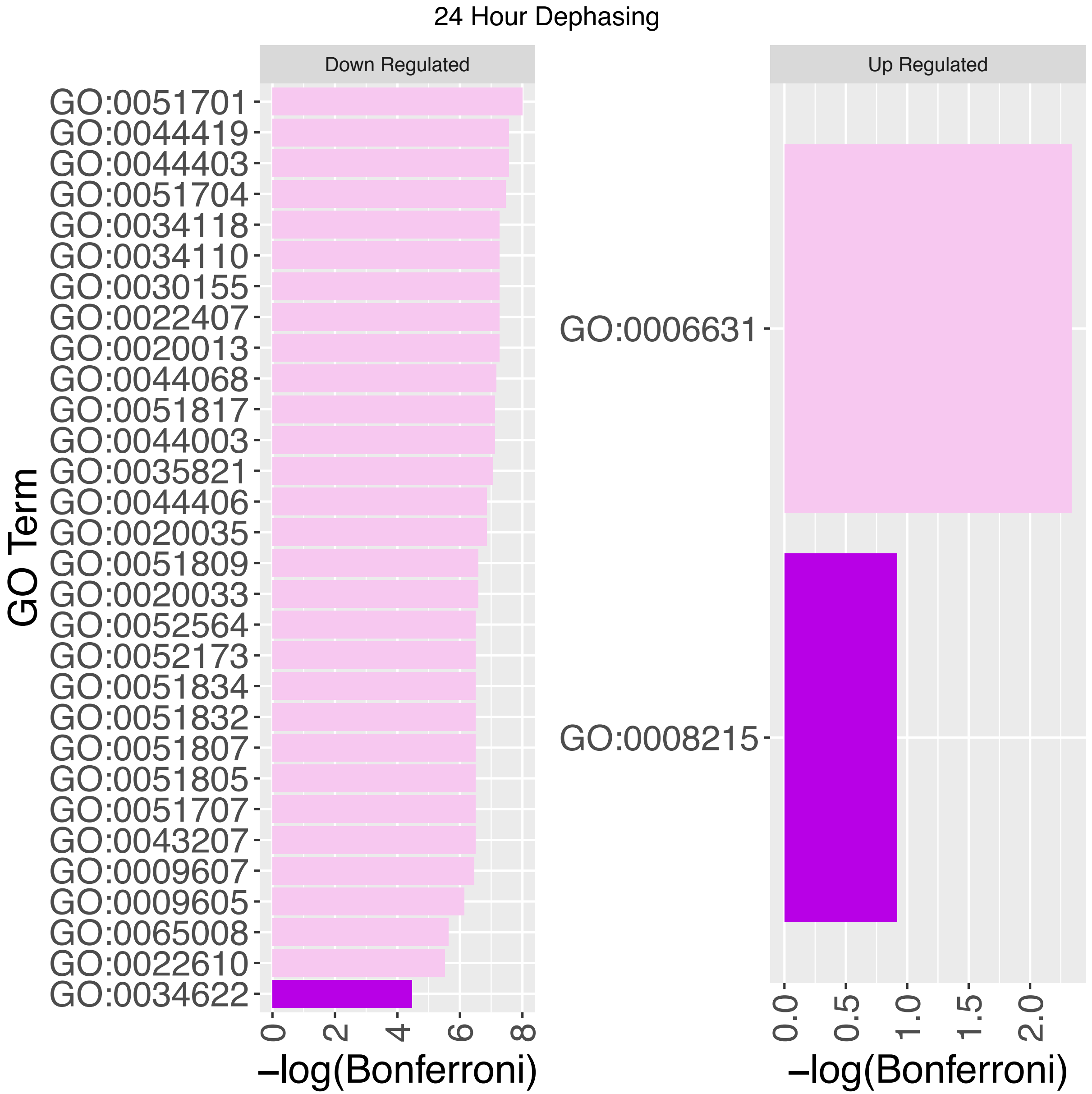

Gene ontology of 24 hour dephasing genes:

The 24 hour dephasing genes form more statistically significant coherent functional groups than randomly sampled genes. Since genes with a shared function are often regulated together this suggests that the DI algorithm is identifying genes that are dysregulated. The down-regulated has an over-representation of genes involved in modifying the host cell.

S2

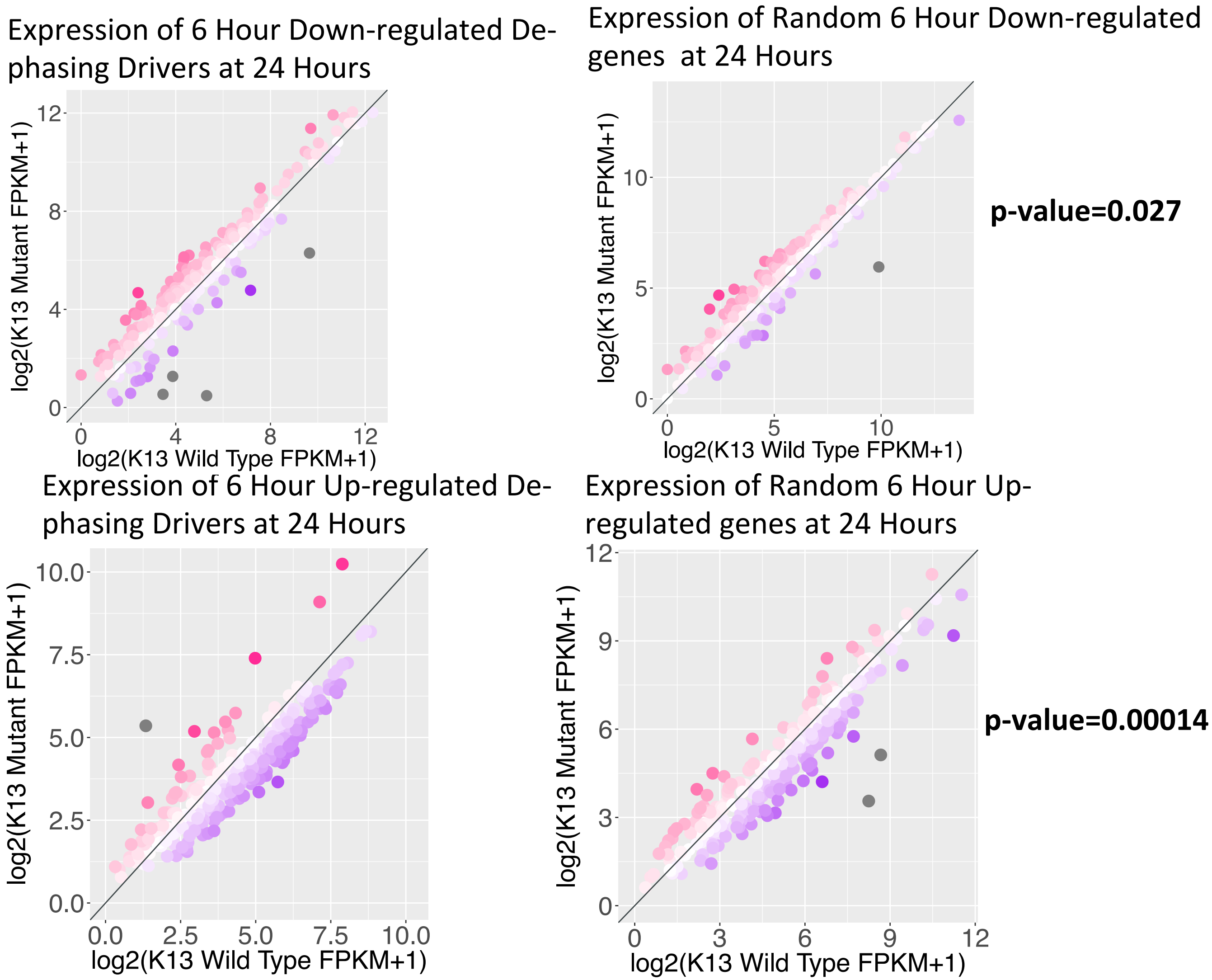

Expression of 6 hour dephasing genes at 24 hours:

6 hour dephasing genes show consistent changes in expression at 24 hours. The decreased relative rank dephasing genes are more likely to be up-regulated at 24 hours than random samples of genes down-regulated at 6 hours. Similarly the 6 hour increased relative rank dephasing genes are more likely to be down-regulated at 24 hours than random samples of genes up-regulated at 6 hours. P-values calculated using a Wilcoxon rank sum test on the log2 fold change compared to random samples of genes that have the same rank difference direction (up or down regulation) as the dephasing set. (S3) The actual fold change distribution and an example random sample and the p-value distributions from 1000 random samplings.

S3

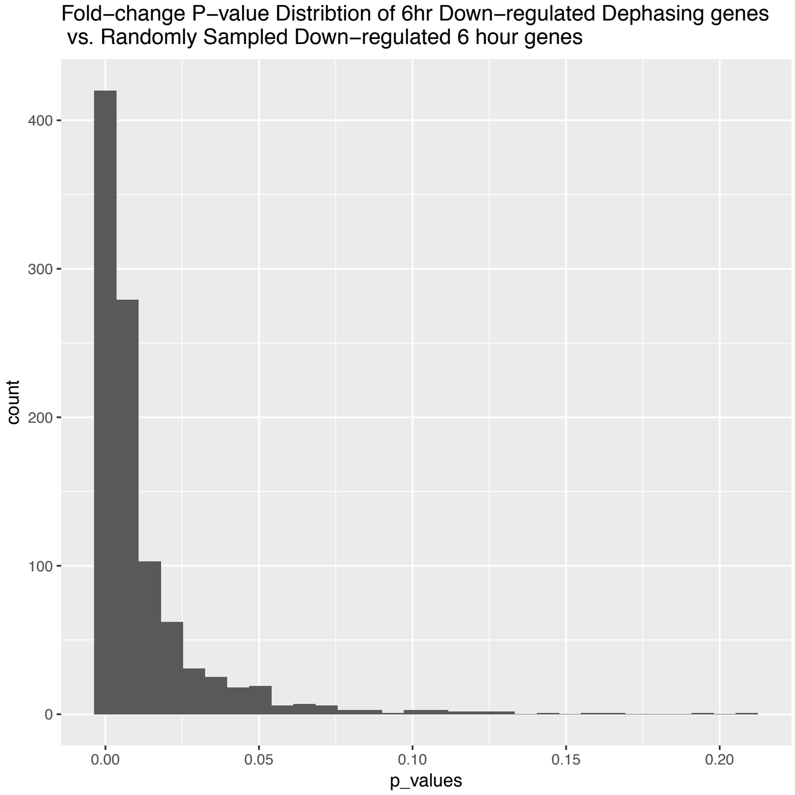

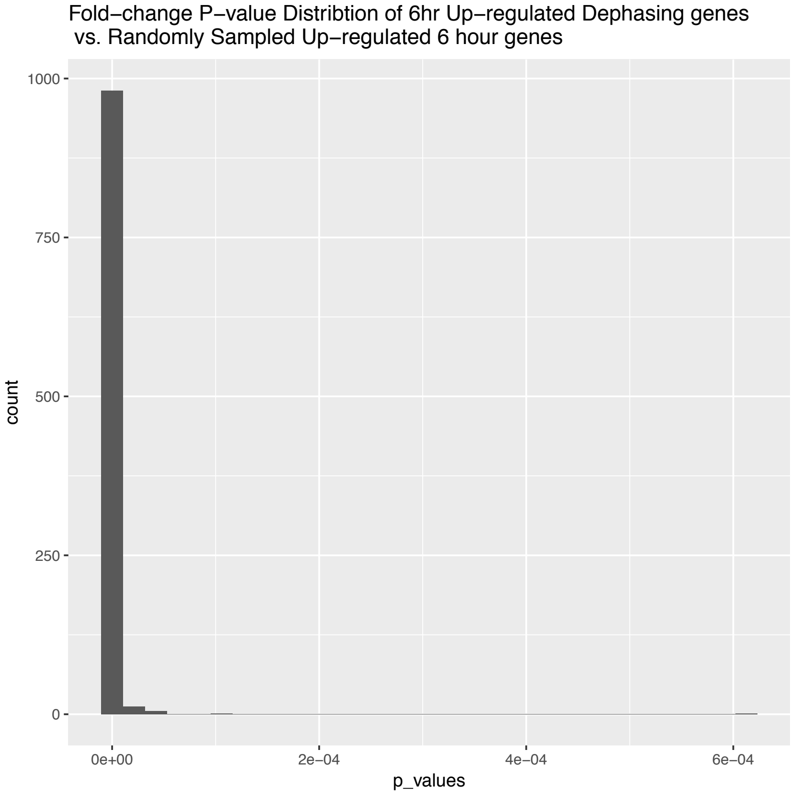

p-value distributions of fold changes for 6 hour dephasing genes at 24 hours vs. random samples:
The p-values were calculated using a Wilcoxon rank sum test of the fold changes of transcript expression between the wild-type and mutant strains for the 6 hour dephasing genes and randomly sampled genes. To test the down-regulated dephasing gene sets the random samples were selected from genes that also had negative rank differences at 6 hours and to test the up-regulated dephasing gene sets the random samples were selected from genes that also had positive rank differences at 6 hours.

S4

A.

6 Hour De-phasing Driver Genes Have Larger Relative Rank Expression Differences Than Expected by Chance

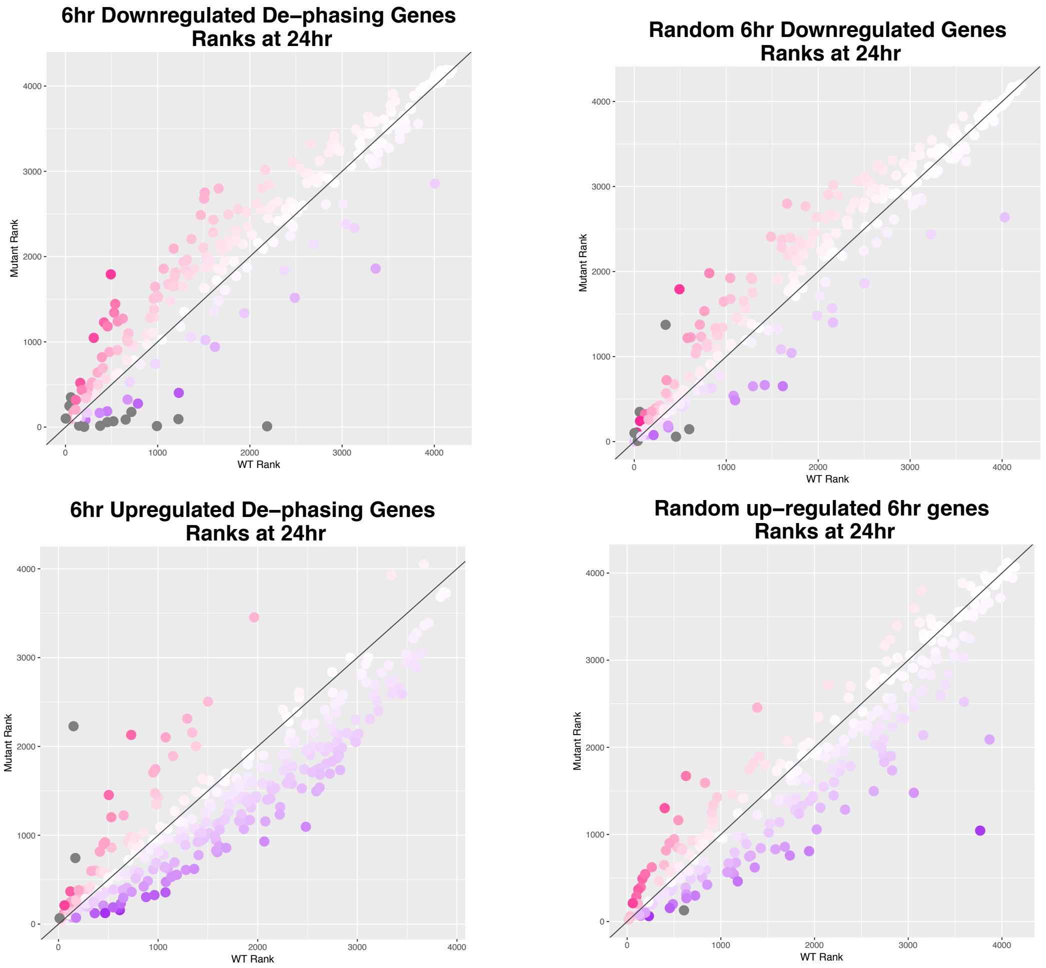

p-value = 0.08868

p-value = 1.263e-05

Dephasing genes show consistent changes in relative rank:

The 6 hour dephasing genes have consistent changes in the relative rank of their expression in relation to K13. The decreased relative rank dephasing genes are more likely to show an increase in the relative rank of their expression at 24 hours , when K13 is up-regulated compared to randomly sampled down-regulated 6 hour genes. Likewise, genes that are increased relative rank 6 hour dephasing genes are more likely to show a decrease in relative rank at 24 hours in the mutant compared to randomly sampled 6 hour up-regulated genes. (S4) The actual relative rank distributions and example random samples. (S5) The p-value distributions that result from 1000 random samplings.

S5

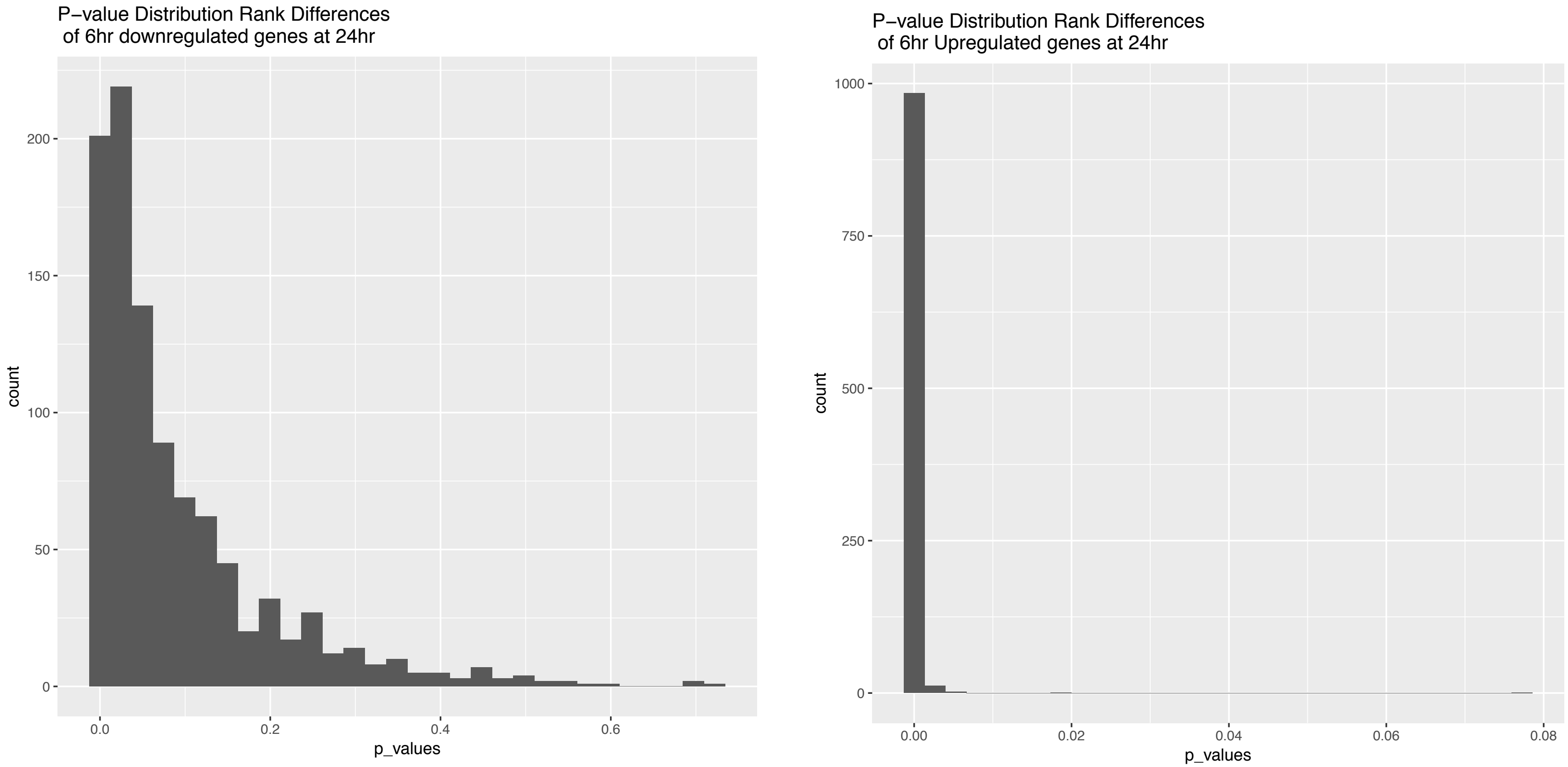

p-value distributions of dephasing gene relative rank differences of 6 hour dephasing genes at 24 hours vs. random samples:

The p-values were calculated using a Wilcoxon rank sum test of the rank differences of transcript expression between the wild-type and mutant strains for the 6 hour dephasing genes and randomly sampled genes. To test the down-regulated dephasing gene sets the random samples were selected from genes that also had negative rank differences at 6 hours and to test the up-regulated dephasing gene sets the random samples were selected from genes that also had positive rank differences at 6 hours.

S6

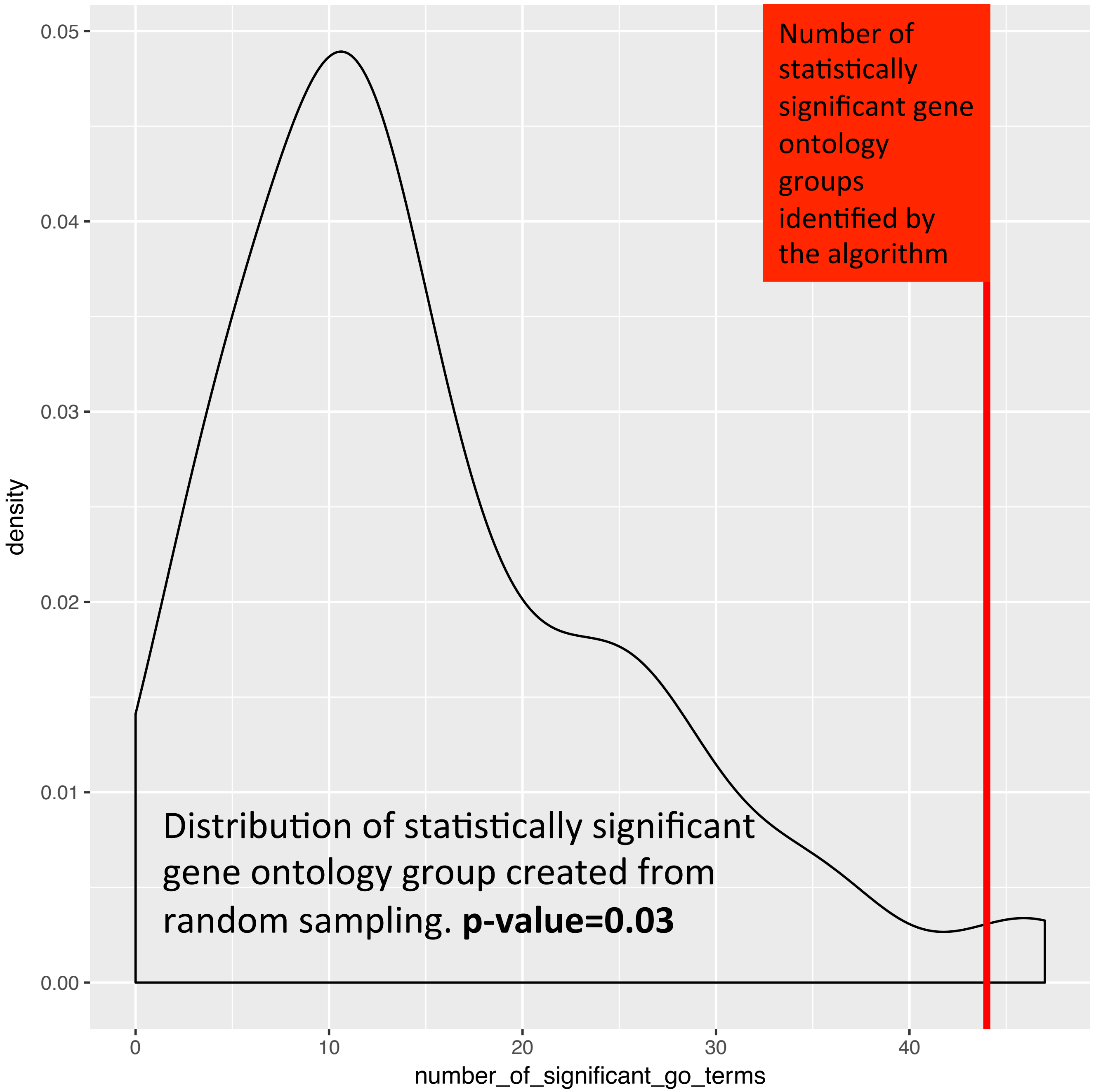
Number of statistically significant gene ontology groups identified by the algorithm
Distribution of statistically significant gene ontology group created from random sampling. **p-value=0.03**
density
0.05
0.04
0.03
0.02
0.01
0.00
0
10
20
30
40
number_of_significant_go_terms

S7

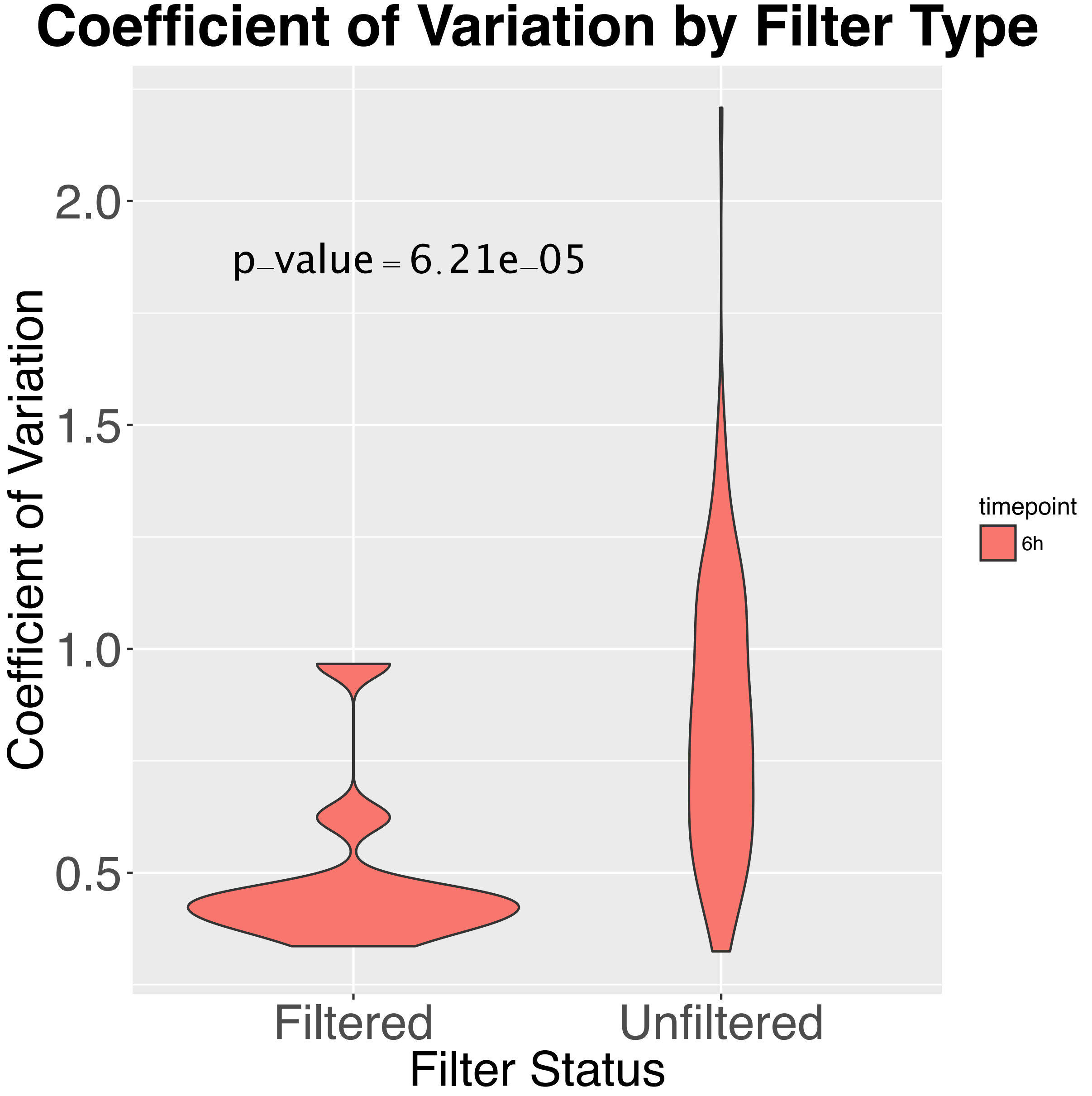

Effect of filtering on DI algorithm performance:

Removing genes with low expression levels (counts per million less than 3) results in genes with lower levels of variation being preferred compared to the unfiltered input (p-value = 6.21e-05 by Wilcox test).
